# Defining the role of NG2-expressing cells in experimental models of multiple sclerosis. A biofunctional analysis of the neurovascular unit in wild type and NG2 null mice

**DOI:** 10.1371/journal.pone.0213508

**Published:** 2019-03-14

**Authors:** Francesco Girolamo, Mariella Errede, Giovanna Longo, Tiziana Annese, Carlotta Alias, Giovanni Ferrara, Sara Morando, Maria Trojano, Nicole Kerlero de Rosbo, Antonio Uccelli, Daniela Virgintino

**Affiliations:** 1 Department of Basic Medical Sciences, Neurosciences and Sense Organs, University of Bari School of Medicine, Bari, Italy; 2 B+LabNet—Environmental Sustainability Lab, University of Brescia, Brescia, Italy; 3 Department of Neurosciences, Ophthalmology, Genetics, Rehabilitation and Child Health, University of Genoa, Genoa, Italy; 4 Center of Excellence for Biomedical Research (CEBR), University of Genoa, Genoa, Italy; 5 Ospedale Policlinico San Martino–IRCCS, Genoa, Italy; Hungarian Academy of Sciences, HUNGARY

## Abstract

During experimental autoimmune encephalomyelitis (EAE), a model for multiple sclerosis associated with blood-brain barrier (BBB) disruption, oligodendrocyte precursor cells (OPCs) overexpress proteoglycan nerve/glial antigen 2 (NG2), proliferate, and make contacts with the microvessel wall. To explore whether OPCs may actually be recruited within the neurovascular unit (NVU), *de facto* intervening in its cellular and molecular composition, we quantified by immunoconfocal morphometry the presence of OPCs in contact with brain microvessels, during postnatal cerebral cortex vascularization at postnatal day 6, in wild-type (WT) and NG2 knock-out (NG2KO) mice, and in the cortex of adult naïve and EAE-affected WT and NG2KO mice. As observed in WT mice during postnatal development, a higher number of juxtavascular and perivascular OPCs was revealed in adult WT mice during EAE compared to adult naïve WT mice. In EAE-affected mice, OPCs were mostly associated with microvessels that showed altered claudin-5 and occludin tight junction (TJ) staining patterns and barrier leakage. In contrast, EAE-affected NG2KO mice, which did not show any significant increase in vessel-associated OPCs, seemed to retain better preserved TJs and BBB integrity. As expected, absence of NG2, in both OPCs and pericytes, led to a reduced content of vessel basal lamina molecules, laminin, collagen VI, and collagen IV. In addition, analysis of the major ligand/receptor systems known to promote OPC proliferation and migration indicated that vascular endothelial growth factor A (VEGF-A), platelet-derived growth factor-AA (PDGF-AA), and the transforming growth factor-β (TGF-β) were the molecules most likely involved in proliferation and recruitment of vascular OPCs during EAE. These results were confirmed by real time-PCR that showed Fgf2, Pdgfa and Tgfb expression on isolated cerebral cortex microvessels and by dual RNAscope-immunohistochemistry/in situ hybridization (IHC/ISH), which detected Vegfa and Vegfr2 transcripts on cerebral cortex sections. Overall, this study suggests that vascular OPCs, in virtue of their developmental arrangement and response to neuroinflammation and growth factors, could be integrated among the classical NVU cell components. Moreover, the synchronized activation of vascular OPCs and pericytes during both BBB development and dysfunction, points to NG2 as a key regulator of vascular interactions.

## Introduction

Oligodendrocyte precursor cells (OPCs) are proliferating elements of the oligodendroglia lineage that remain in the adult brain at the end of oligodendrogenesis as a unique type of potentially self-renewing glia and generate oligodendrocytes throughout life [1, 2]. Similarly to what occurs during central nervous system (CNS) development, in adult CNS, OPCs express high levels of nerve/glial antigen 2 (NG2) chondroitin sulfate proteoglycan 4 constitutively, and are often indicated as NG2-glia, the two terms being interchangeable in the literature [3]. OPCs/NG2-glia are the fourth glial type in adult CNS, making up 5–8% of the total glia in grey and white matter, where these cells develop functional relations at synapses and Ranvier’s nodes [4, 5]. After myelin damage due to white matter traumatic injury or neuroinflammation, OPCs/NG2-glia reload a proliferative phenotype and differentiate, becoming a potential source for replacement of degenerating oligodendrocytes [6–9]. As described previously in cerebral cortex of mice affected with experimental autoimmune encephalomyelitis (EAE), OPCs proliferate during EAE and are present in close vicinity to damaged blood-brain barrier (BBB) microvessels [10]. The same model of EAE repeated in NG2 knock-out mice leads to a milder disease characterized by a less inflammatory profile of pathogenic Th1 lymphocytes, reduced inflammatory infiltrate, and preserved BBB function [11]. Overall, these data suggest that if vascular OPCs are actually members of the neurovascular unit (NVU), they could be involved in the alteration of BBB function during neuroinflammation, and NG2 could interact with endothelial cells engaging the tight junctions (TJs) in the disease response. This idea is supported by recent data on OPC/vessel mutual relationships [12, 13] and by growing evidence of the importance of NG2 proteoglycan molecular interactions and biological functions [14, 15]. During neurohistogenesis, OPCs migrate along brain microvessels according to their tissue fate map [12], and are involved in vessel sprouting, as well as in the establishment of a proper brain vascular network [13]. The structure of NG2, as a type 1 integral membrane glycoprotein, enables it to work in a *cis* modality as a self-sensor/transducer, which interacts through the cytoplasmic PDZ (PSD-95, DISC-large, ZO-1)-binding domain with scaffolding proteins to control cell migration, but also as a non-self-regulator through *trans* interactions with the accompanying cells established by its extracellular domain [16, 17]. NG2 *trans* activity has been extensively studied in the vascular system, where the proteoglycan, expressed by immature pericytes, influences endothelial cell adhesion, spreading and migration [16]. In this study, we used immunoconfocal morphometry and TJ protein immunohistochemistry to quantify vascular OPCs/NG2-glia (hereinafter referred to as OPCs) and investigate whether a vascular contingent of NG2-bearing OPCs may contribute to TJ organization in the cerebral cortex of developing and adult naïve wild-type (WT) and NG2 knock-out (NG2KO) mice, as well as after EAE induction in WT and NG2KO mice. The expression of the growth factors revealed by immunohistochemistry was also analysed by real time-PCR on isolated cortex microvessels and by dual RNAscope immunohistochemistry/in situ hybridization (IHC/ISH) on cortex section from EAE-affected WT mice. The results demonstrate that OPCs are a dynamic, integral cell component of the developing and adult NVU, and support the hypothesis of NG2 as the molecule involved in TJ regulation during BBB development and dysfunction.

## Materials and methods

### Mice

Wild-type (WT) C57Bl/6J mice were purchased from Harlan (Bresso, Italy). The NG2KO mice were obtained, monitored regularly for the insertion leading to NG2 gene disruption, and maintained as described in detail previously [11]. All applicable international, national, and/or institutional guidelines for the care and use of animals were followed (Italian Law Decree 4 March 2014, n. 26, legislative transposition of Directive 2010/63/EU of the European Parliament and of the Council of 22 September 2010 on the protection of animals used for scientific purposes). The research protocol was approved by the Ethics Committee for Animal Experimentation of the University of Genoa, Italy.

### EAE induction

Chronic EAE was induced by active immunization with myelin oligodendrocyte glycoprotein peptide spanning amino acids 35–55 in female WT and NG2KO mice (8 weeks of age, weighing 18.5 ± 1.5 g) and mice were monitored for clinical manifestations as described in detail previously [18]. Mice were sacrificed by gradual-fill CO_2_ exposure at P76 and P96, i.e. 20 and 40 days post-immunization (dpi), respectively. A score of 4 or a 25% weight loss is considered to be a humane endpoint and our animal ethical policy in these cases is euthanasia. However, the protocol followed in this study for EAE induction rarely, if ever, reaches a score of 4 in our hands. Actual induction of EAE by subcutaneous immunization in the flanks does not result in pain or specific discomfort or distress for the mice. There are no obvious manifestations of pain in mice affected with EAE, which, despite hind-limb paralysis at the highest score tolerated, are still active. Pain cannot be excluded, however, as neuropathic pain is reported by 60–70% of patients with multiple sclerosis. Nevertheless, the use of anti-inflammatory and analgesic agents is not recommended in EAE as it modulates the disease course and immunological status of the mice.

### Immunohistochemistry

WT and NG2KO mice at postnatal day 6 (P6), and adult naïve and EAE-affected WT and NG2KO mice at 20 and 40 dpi, were transcardially perfused with 100–150 ml of 2% paraformaldehyde (PFA) and 0.2% glutaraldehyde PBS solution, under deep anaesthesia (ketamine/xylazine cocktail, 90 mg and 4.5 mg/kg, respectively) by intraperitoneal injection. Whole brains were removed and post-fixed by immersion in the same fixative at 4°C for 4 h, washed in PBS overnight at 4°C, and stored in 0.02% PFA in PBS at 4°C. Using a vibrating microtome (Leica Microsystem), serial sagittal sections (30/35-μm thick) evenly spaced at 200 μm intervals, were cut from each hemisphere to allow the analysis of the entire antero-posterior extension of the cerebral cortex. The sections, in a range of 60 to 120 sections/hemisphere, were stored in a multiwell archive as free-floating sections in PBS at 4°C. The archived sections were submitted to immunostaining to ascertain the presence of demyelinating lesions, and the adjacent sections were then utilized for confocal morphometry (see for details [6]). Briefly, after permeabilization (0.5% Triton X-100 in PBS), free-floating sections were incubated with single or combined primary antibodies, overnight at 4°C, and with appropriate secondary antibodies (Table 1), for 45 min at RT, and then counterstained with TO-PRO-3 diluted 1:10k in PBS (Invitrogen). Finally, the sections were collected on Vectabond treated slides (Vector) and coverslipped with Vectashield (Vector). Negative controls were prepared by omitting the primary antibodies and mismatching the secondary antibodies. Sections were examined under Leica TCS SP5 confocal laser scanning microscope (Leica Microsystems) using a sequential scan procedure. Confocal images were taken at 0.35 μm intervals through the z-, x-, and y-axes of the section, with 40x and 63x oil lenses.

**Table 1:** List of primary and secondary antibodies (Abs) used in immunohistochemistry and immunoconfocal morphometry.

**Primary Abs**	**Host IgG**	**Concentration** **(μg/μl)**	**Supplier**	**Catalogue No**
CD45	Rat IgG_2_	1	Novus Biological	NB110-93609
Iba1	Gt IgG	5	Abcam	ab5076
NG2	RbIgG	5.5	Millipore	AB5320
GFAP	Mo IgG_1_	6,7	V.B. Novocastra	NCL-GFAP-GA5
CD31	Rb IgG	0.6	Abcam	ab28364
PDGFRα	Rat IgG_2ak_	7.1	Millipore	CBL1366
PDGFRβ	Mo IgG_2a_	1.0	Calbiochem	GR23L
CD13 FITC	Rat IgG	10.0	BD Pharmigen	558744
Claudin-5	Mo IgG_1_	16,6	Thermo Fisher	18–7364 4C3C2
Occludin	Rb IgG	5.0	Thermo Fisher	71–1500
Laminin	Rb IgG	3.3	Sigma	L9393
Collagen type VI	Rb IgG	10.0	Abcam	ab6588
Collagen type IV	Rb IgG	12.5	Acris	R1041
VEGF-A	Rb IgG	1,4	Immunological Science	AB90040
VEGFR2	Rb IgG	NA	NovusBiologicals	NB100-627
FGF2	Rb IgG	11.1	Santa Cruz Biotecnology	sc-79
FGFR1	Rb IgG	0.7	Santa Cruz Biotecnology	sc-121
PDGF-AA	Rb IgG	25.0	Abcam	ab125268
TGF-β	Mo IgG_1_	2.5	Chemicon	MAB1032
**Secondary Abs and reagents**		**Concentration (μg/μl)**	**Supplier**	**Catalogue No**
Goat anti mouse IgG_1_ Alexa 568		6.6	ThermoFisher	A21124
Goat anti mouse IgG_1_ Alexa 633		6.6	Invitrogen	A21126
Goat anti rat Alexa 555		2.5	Invitrogen	A21434
Donkey anti goat Alexa 568		6.6	Invitrogen	A11057
Goat anti mouse IgG_2a_ Alexa 633		6.6	ThermoFisher	A21136
Goat anti-rabbit Alexa 488		6.6	Invitrogen	A11070
Goat anti mouse IgG_1_Alexa 488		6.6	Invitrogen	A11001
Goat anti mouse IgG_2a_ Alexa 488		6.6	ThermoFisher	A21131
Goat anti-rabbit Alexa 568		6.6	Invitrogen	A11011
Biotinylated goat-anti rabbit		3.75	Vector	BA-1000
Streptavidin-Alexa 488		6.6	Invitrogen	S-11223
Streptavidin-Alexa 555		6.6	Invitrogen	S-21381

NA not available

### FITC-dextran experiments

A solution of heparin (100 Units/kg) containing Fluorescein isothiocyanate–dextran 70-kDa (5 mg/ml; FITC-dextran 70, Sigma-Aldrich), as a fluorescent probe to evaluate BBB-endothelial cell permeability, was injected into the tail vein of adult naïve, WT (n = 5) and NG2KO (n = 5) mice, and EAE-affected mice, WT (n = 5) and NG2KO (n = 5). Four minutes after the end of the injection, the mice were anesthetized, and brains were collected and fixed in 2% paraformaldehyde and 0.2% glutaraldehyde in PBS solution. 30/35-μm-thick sections were immunolabelled with antibody anti-PDGFRα (Table 1), as described above, counterstained with TO-PRO-3 and analyzed by confocal laser microscopy.

### Laser confocal microscopy morphometry

**Fig 1:**
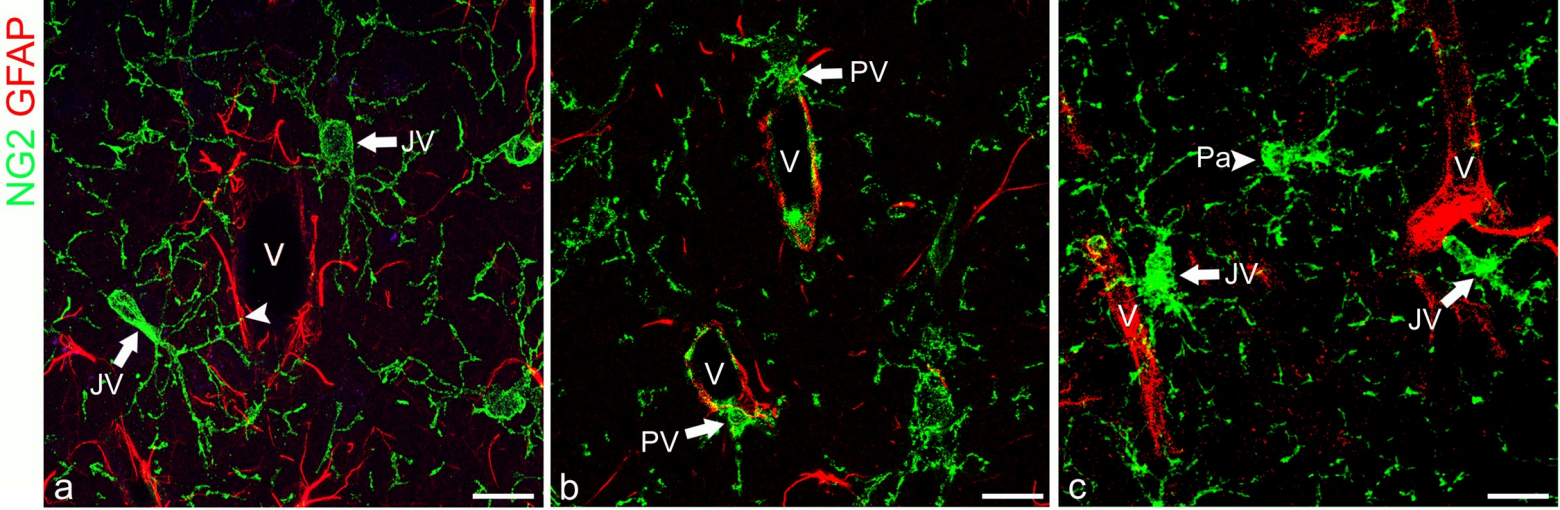
A subpopulation of NG2^+^ OPCs resides on microvessels. **a-c** The processes of NG2^+^juxtavascularOPCs (JV, arrows) are in contact with the vessel wall (V). **A** In particular, there is a JV OPC process crossing a GFAP^+^endfoot through a small hole (arrowhead). **B** Two NG2^+^ perivascular OPCs (PV; arrows) apposed on the abluminal side of microvessels (V) with their bodies and processes projecting around the vessel wall and in the surrounding neuropil. **c** A representative parenchymal OPC (Pa, arrowhead) virtually unassociated with vessels because none of its processes reaches vessel wall (V) in this image stack (20 μm-thick) is together with two JV OPCs (JV, arrows). Scale bars, 30 μm.

Quantitative assessment was carried out on 4 sections per brain from WT (n = 4) and NG2KO (n = 4) mice at P6, and from EAE-affected WT (at 20 dpi, n = 5, clinical score ranging from 2.5 to 3.5; at 40 dpi, n = 5, clinical score ranging from 2.5 to 3.0) and NG2KO mice (at 20 dpi, n = 5, clinical score ranging from 1.5 to 3.0; at 40 dpi, n = 5, clinical score ranging from 1.5 to 2.0; for mean clinical scores see ref.11) and adult age-matched naïve WT (n = 5) and NG2KO (n = 5) mice, by computer-aided morphometric analysis using the Leica Confocal Multicolor Package (Leica Microsystems) and ImageJ (NIH) software. The microvessels included in the morphometric analysis ranged from capillaries (4–6 μm in diameter) to small arterioles and venules (10–20 μm in diameter). The numbers of capillary sprouting points, pericytes, and OPCs were interactively counted (Cell counter ImageJ) and the laminin area fraction and thickness were measured on serial 0.35 μm optical sections across 30–35 μm-thick projected Z-stack images of randomly chosen fields (10 fields, total area = 150000 μm^2^ each) from nearly identical regions of cerebral cortex (4 sections/brain). NG2^+^ or PDGFRα^+^ OPCs were categorized as: perivascular (PV), with the cell body in contact with the vessel wall; juxtavascular (JV), with cell processes in contact with the vessel wall; parenchymal (Pa), when virtually unassociated with vessels (Fig 1A–1C). The accurate identification of OPCs in each of the described categories, JV, PV, and Pa, primarily depended on the examination of single optical planes of confocal z-stacks at intervals of 0.35 μm. The 'reference vascular volume' for each vessel included the surrounding tissue at a radiate distance of 15 μm from the vessel wall. The rationale for this parameter was the distance range calculated for the JV OPCs (14.67 ± 4.91 μm on 188 measurements of the minimal distance between the nuclear region of NG2^+^ and PDGFRα^+^ JV OPC and the contact point of its process and the nearest vessel wall). The vascular volume and the corresponding value were modified only to include the JV OPC, whose processes extend beyond the reference distance range. The density of OPCs was normalized to the same cumulative vessel length (CVL = multiple of 100 μm of vessel length), excluding vessels with a diameter >30 μm. The density of doublet OPCs, regarded as a sign of OPC proliferation, was normalized to either CVL (100 μm) or volume (10^6^ μm^3^). NG2^+^ or PDGFRβ^+^ or CD13^+^ pericytes were recognized as vessel-embracing cells, counted, and the data reported as percentage or density (number of pericytes/CVL). We used laminin staining to detect modifications of vessel basal lamina. The laminin area fraction was measured on threshold images as number of positive pixels on total pixel number. Laminin thickness was measured at three points for each transverse-oriented vessel profile on single confocal optical planes. After treatment of mice with the permeability marker FITC-dextran 70 kDa, leaky BBB-microvessels were localized by fluorescent dextran extravasation and interactively counted.

### Isolation of cerebral cortex vessels and real time-PCR

Brains removed from naïve WT (n = 6) and EAE-affected WT (n = 6) mice, and stored in 0.02% PFA in PBS at 4°C (see Immunohistochemistry section), were washed two times in PBS for 2 min each and cerebral cortices were dissected under a stereomicroscope. The cortices from each experimental group were then dissociated using the Adult Brain Dissociation Kit (130-107-644, Miltenyi Biotec, Germany), optimized to obtain isolated microvessels. Briefly, small amounts of cortex (<100 mg) were put in MACS C Tubes (130-093-237, Miltenyi Biotec) containing Enzyme mix 1 and Enzyme mix 2. Tightly closed C Tubes were attached upside down onto the sleeve of the gentleMACS OctoDissociator with Heaters. Samples were dissociated applying the protocol 37C_ABDK_02 optimized for vessel isolation. Then tubes were centrifuged at 2000g for 2 min and the samples were filtered on MACS SmartStrainers 30 μm (130-098-458, Miltenyi Biotec). Filtrates were centrifuged at 2000g for 10 min at +4°C and pelleted blood vessels were resuspended in PBS in multiwell plates and stored at 4°C until confocal immunofluorescence or real time-PCR analysis. The whole pellet samples were processes for immunohistochemistry (see Immunohistochemistry section) with the following Abs: anti-CD31 alone and anti-CD31 combined with anti GFAP (Table 1) overnight at 4°C, detected by appropriate fluorophore-conjugated secondary antibodies for 45 min at RT, and then counterstained with TO-PRO-3 diluted 1:10k in PBS (Invitrogen). Finally, the pellet samples were transferred on Vectabond treated slides using a glass pipette and coverslipped with ProLong Diamond Antifade Mountant (Thermo Fischer Scientific, Cat No P36961). Sections were examined under Leica TCS SP5 confocal laser scanning microscope (Leica Microsystems) using a sequential scan procedure.

For real time-PCR, total RNA was extracted from cerebral cortex isolated microvessels, using commercially available RecoverAll Total Nucleic Acid isolation kit (Ambion, Life Technologies, Inc., Austin, TX, USA), skipping the step of deparaffinization. RNA integrity was verified by OD260/OD280 nm measurement (absorption ratio >1.95), and 1 μg was reverse-transcribed using iScript cDNA Synthesis Kit (Bio-Rad Laboratories, Hercules, CA, USA) according to the manufacturer’s instructions. For the detection of Fgf2, Pdgfa and Tgfb expression, cDNA was amplified with iTaq Universal SYBR Green Supermix (Bio-Rad Laboratories, Cat No 172–5124) and with specific Bio-Rad Prime PCR (Bio-Rad Laboratories: Fgf2, qMmuCID0015817; Pdgfa, qMmuCID0022342; TGF-β, qMmuCID0017320), using the Chromo4 real time-PCR Detection System (Bio-Rad Laboratories). Samples were normalized to mouse β-Actin gene expression (qMmuCED0027505; Bio-Rad Laboratories). All the experiments were performed in duplicate with polymerase activation for 30 sec at 95°C, cDNA denaturation for 15 sec at 95°C, annealing and extension for 30 sec at 60°C for 35 cycles. The melting curve analysis was performed at 65°-95°C intervals with 0.5°C temperature increases per reading step. The fold-change values were calculated with the comparative Ct method (2^-ΔΔCt^). Results are given as mean±SD.

### Dual RNAscope-immunohistochemistry/in situ hybridization (IHC/ISH)

Brain sections from EAE-affected mice (n = 2) were collected from the multiwell archive of sections (see Immunohistochemistry section) and washed four times in PBS for 10 min each. After adhesion on polylysine slides (Menzel-Glaser, Braunschweig, Germany) by drying for 10min at RT, the sections were incubated with anti-GFAP, anti-CD31, or anti-PDFGRα primary antibodies (listed in Table 1) overnight at 4°C, detected by Alexa 488-conjugated secondary antibodies for 45 min at RT, and subsequently processed for RNAscope Technology (Advanced Cell Diagnostic, ACD, Inc.; Hayward, CA, USA) as follows: the sections were washed three times in PBS for 10 min each, incubated in Pretreatment 1 (H_2_O_2_) for 60 min at RT and then rinsed four times in PBS for 1min each. The sections were dried for 1 hour at RT, then incubated in Pretreatment 2 target retrieval reagent (pre-treatment kit Cat. No 322330, ACD) for 7 min at 99–104°C, washed in H_2_O for 1min twice and then dried for 10 min at RT. The slides were dipped in 100% ethanol (EtOH) and air-dried and the sections demarcated by a hydrophobic fence using a ImmEdge Hydrophobic Barrier Pen (ACD, Cat. No 310018). The hydrophobic barrier was allowed to completely dry for 1 hour at RT. Then, the sections were incubated in Pretreatment 3 (ACD, protease, pretreatment kit Cat. No 322330) for 15min at 40°C in the EZ Hybridization oven (ACD, Cat. No 310012) using the humidity control tray and slide rack (ACD, Cat. No 310014) and washed four times in H_2_O for 1 min each. Afterwards, the sections were incubated with Vegfa (ACD, Cat. No 436961) or Vegfr2 probes (ACD, Cat. No 414811) (4 drops/section) for 2 hours at 40°C and then washed two times in 1x wash buffer (ACD Cat. No 310091) for 2 min each. The following amplification (Amp) and detection steps were performed using the RNAscope 2.5 HD Reagent Kit-RED (ACD, Cat No 322350) according to the following incubation steps: Amp1 for 30 min at 40°C, Amp2 for 15 min at 40°C, Amp3 for 30 min at 40°C, Amp4 for 15min at 40°C, Amp5 for 30 min at RT in HybEZ humidity control tray and slide rack (ACD) to maintain humidity, Amp6 for 15 min at RT. After each incubation step, the sections were washed twice in wash buffer for 2 min. The ISH signal was detected by incubating the slides in a mixture of Fast-Red-A solution and Fast-Red-B solution at a 1:60 ratio for 10 min at RT. The slides were washed in H_2_O twice for 2 min, counterstained with Sytox Green (diluted 1:5k in PBS for 10 min; Thermo Fisher Scientific), and the sections were coverslipped with ProLong Diamond Antifade Mountant (Thermo Fischer Scientific, Cat No P36961). Sections were examined under Leica TCS SP5 confocal laser scanning microscope (Leica Microsystems) using a sequential scan procedure to detect the fluorescence signal of both Alexa 488-secondary Abs and Fast-Red-A-probes.

### Statistical analysis

All data, expressed as mean value ± SD (standard deviation), were statistically analyzed using Student’s t-test, one-way ANOVA and the Bonferroni post-test (GraphPad Prism, GraphPad Software, Inc.). Raw, numeric data utilized for statistical analysis are shown in Supporting information (S1 Excel file). For real-time PCR analysis, the statistics were performed with two-way ANOVA analysis and Bonferroni post-hoc tests to compare replicates using GraphPad Prism 5.01. *P*<0.05 was considered as the limit for statistical significance.

## Results

### An in-depth analysis of vascular NG2-expressing cells by immunoconfocal morphometry

**Fig 2:**
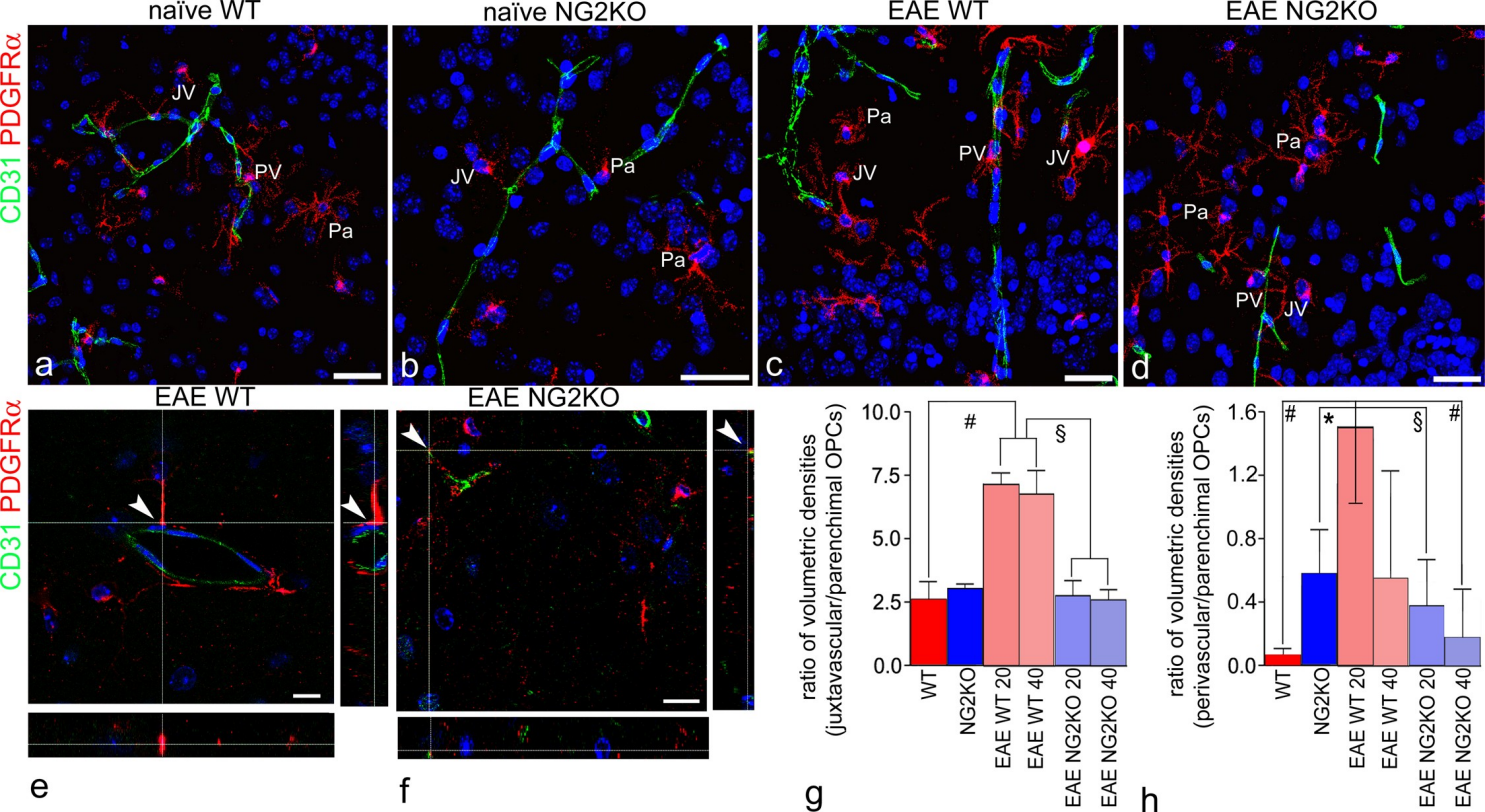
PDGFRα/CD31-based confocal morphometry identifies vessel-associated OPCs. **a**-**d** Representative confocal images (stacks of xy planes on the z-axis; projection images) of cerebral cortex sections double-immunolabelled for CD31, a marker for endothelial cells, and PDGFRα, used to identify and count juxtavascular (JV), perivascular (PV), and parenchymal (Pa) OPCs. **e**, **f** Representative xy single planes and their respective xz (bottom) and yz (right) planes, used to disclose single OPC/endothelial cell contacts (arrowheads). **g, h** Bar charts show the ratio between JV OPC and Pa OPC volumetric density (**g**) and the ratio between PV OPC and Pa OPC volumetric density (**h**); both the parameters demonstrate a significant increase of JV and PV OPCs in EAE WT mice at 20 dpi; no significant differences are observed in the JV OPC density between naïve and EAE NG2KO mice. Data for both panels **g** and **h** are presented as mean ± SD; *p< 0.05, ^§^*p*< 0.01, ^#^*p*< 0.001. n = 5. EAE-affected WT mice (at 20 dpi:clinical score range = 2.0–3.5, mean number of counted JV OPCs 270 per mouse. At 40 dpi: clinical score range = 2.0–3.0 mean number of counted JV OPCs = 230 per mouse), EAE-affected NG2KO mice (at 20 dpi: clinical score range = 1.5–2.5 mean number of counted JV OPCs = 108 per mouse. At 40 dpi: clinical score range = 1.0–2.0 mean number of counted JV OPCs = 203 per mouse). Nuclear counterstaining with TO-PRO-3. Scale bars, **a**-**d** 50 μm and **e**, **f** 10 μm.

The antibodies utilized for morphometric analysis (Table 1) included: NG2 and platelet-derived growth factor receptor α (PDGFRα), as markers of OPCs; CD13, an aminopeptidase constitutively expressed by mature pericytes; NG2 and platelet-derived growth factor receptor β (PDGFRβ) as markers for activated pericytes; CD45 and Iba1 for revealing monocytes/macrophages, which in pathological contexts could transiently express NG2 [19–21]. NG2^+^ monocytes/macrophages, associated with cortex microvessels, were rarely seen and, therefore, they were excluded by a morphometric evaluation (S1A–S1F Fig). OPCs immunolocalized by both NG2 and PDGFRα, were classified according to their position with regard to the vessel wall, as perivascular (PV), when the cell body was in contact with the vessel wall and juxtavascular (JV), when only the cell processes contacted the vessels; OPCs scattered in the cortex, and virtually not associated with the vessel wall, were classified as parenchymal (Pa) OPCs (Fig 1A–1C). Because quantitative evaluation of cell-vessel contacts could be impaired by technical reasons, our morphometric analysis relied on high resolution confocal microscopy applied to vibratome-cut sections (about 30–35 μm thick), to ensure a well-preserved tissue structure and organization. Each vascular field, recognized on the whole sagittal prospect of cerebral cortex, was recorded as single optical planes at intervals of 0.35 μm and as stacks of xy planes on the z-axis (Fig 2A–2D), xz planes through the y axis, and yz planes through the x axis (Fig 2E and 2F). To make possible a comparative analysis, of the selected morphometric parameters between WT and NG2KO mice, morphometry was firstly applied to NG2/PDGFRα and NG2/PDGFRβ stained sections from naïve and EAE-affected mice. The results showed, for each of the tested pairs of molecules, an excellent overlapping of the immunostaining, thus validating their reliability as alternative morphometry markers and confirming previous studies that unequivocally identify the same glial population with NG2 and PDGFRα [22, 23] and pericytes in an activated status with NG2 and PDGFRβ [3].

### PDGFRα/CD31-based confocal morphometry identifies vessel-associated OPCs

**Fig 3:**
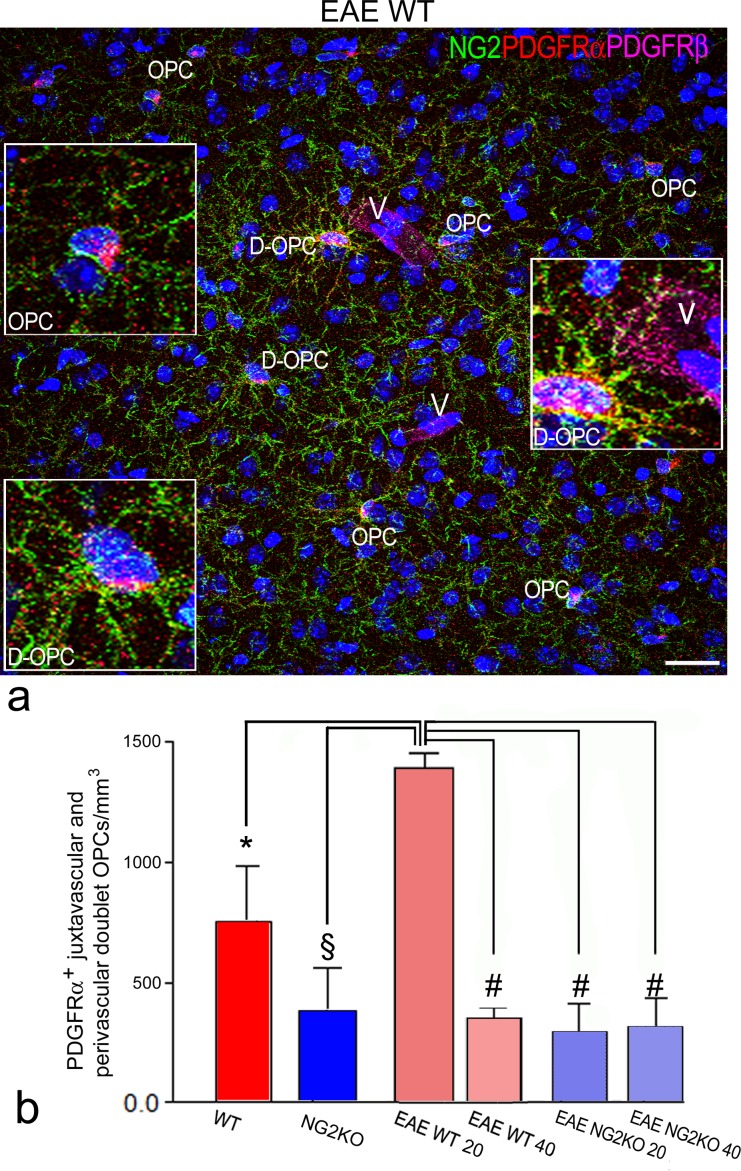
Increase in doublet OPCs in EAE suggests the proliferation of vascular OPCs. **a** Representative confocal image from an EAE-affected WT mouse (clinical score = 2.5) of multiple immunostaining for NG2, PDGFRα and PDGFRβ shows doublet OPCs (D-OPC) reactive for NG2 and PDGFRα, which also contact the microvessel wall revealed by PDGFRβ^+^pericytes (V), together with NG2^+^/PDGFRα^+^processes of OPCs (OPC). Zoomed insets (3x) show details of the vessel-contacting D-OPC (right zoom), a parenchymal D-OPC (left lower zoom), and a parenchymal OPC (left upper zoom). **b** The bar chart shows that the total value of JV and PV D-OPCs, quantified by confocal morphometry on brain sections immunolabelled with anti-PDGFRα antibody, increases in EAE-affected WT mice at 20 dpi compared with all the other experimental groups. Data are presented as mean ± SD cell number/10^6^ μm^3^; n = 5; *p<0.05, ^§^*p*< 0.01, ^#^*p*<0.001. EAE WT mice (at 20 dpi: clinical score range = 2.0–3.5, mean number of counted JV doublet OPCs = 25 per mouse. At 40 dpi: clinical score range = 2.0–3.0, mean number of counted JV doublet OPCs = 12 per mouse), EAE NG2KO (at 20 dpi: clinical score range = 1.5–2.5, mean number of counted JV doublet OPCs = 8 per mouse. At 40 dpi: clinical score range = 1.0–2.0, mean number of counted JV doublet OPCs = 16 per mouse). Nuclear counterstaining with TO-PRO-3. Scale bar, 50 μm.

Based on this preparatory work, double-staining for PDGFRα and CD31 was carried out on cerebral cortex sections from WT and NG2KO mice at postnatal day 6 (P6), and from adult naïve and EAE-affected WT and NG2KO mice (n = 5 per group), at 20 and 40 days post-immunization (dpi), in view of the different behaviour of OPCs during the disease course [6] (Table 2 and Fig 2A–2H). When density of JV and PV OPCs was measured according to CVL (cumulative vessel length), which corresponds to multiples of 100 μm of vessel length, the highest values in healthy mice were obtained in WT mice at P6 (JV = 0.89±0.22 and PV = 0.10±0.06; Table 2). In adult naïve WT mice, JV and PV OPCs diminished to a level (JV = 0.47±0.13 and PV = 0.02±0.01) that established the baseline of vascular OPCs in the adult normal brain (Table 2 and Fig 2A). In NG2KO mice at P6 and in adulthood, these values were both significantly reduced (P6 mice, JV = 0.48±0.21 and PV = 0.05±0.06; adult mice, JV = 0.31±0.08 and PV = 0.005±0.003; Table 2 and Fig 2B). The proliferative response of OPCs to EAE-associated neuroinflammation we observed in the cerebral cortex of WT mice [6], was reflected by the increase of both JV and PV OPCs at 20 dpi (JV = 0.79±0.14 and PV = 0.23±0.11), a value that decreased at 40 dpi, when the vascular density of OPCs reverted to the baseline values (JV = 0.59±0.13 and PV = 0.05±0.07; Table 2 and Fig 2C). The density values of OPC *per* CVL obtained in EAE-affected NG2KO mice, at both 20 and 40 dpi, although increased, did not significantly change compared to naïve WT and NG2KO mice (Table 2 and Fig 2D). The relative proportion of each OPC subset, expressed as a percentage of total OPC number, revealed a prevalence of JV OPCs over both PV and Pa OPCs in EAE WT mice (Table 2). This datum was confirmed by quantification of ratios of JV OPC/Pa OPC volumetric density and PV OPC/Pa OPC volumetric density, that were both significantly increased in EAE WT mice (Fig 2G and 2H). As a mitotically active population, reacting rapidly to CNS damage, OPCs have unique proliferation profile, identified by symmetrical pairs of BrdU^+^/NG2^+^ cells that remain close to one another (doublet OPCs) over a period of 10 days and account for approximately 70% of the BrdU^+^cells detected after a short pulse of BrdU [5, 15, 24]. As a hallmark of OPC proliferation, doublet OPCs were morphometrically counted to find out the mitotically active proportion of OPCs in both the vascular and parenchymal subsets (Table 3). Vascular doublet (JV+PV) density, calculated according to cell number *per* CVL and *per* perivascular volume (cortex volume unit equal to 1 mm^3^) parameters, was compared with Pa doublet OPC density (Table 3 and Fig 3A and 3B). The results showed a peak in vascular doublet linear and volume densities in EAE-affected WT mice at 20 dpi (Table 3 and Fig 3B). When compared with all the other groups, this was similar only to the density obtained in P6 WT mice (Table 3), while both values were diminished in NG2KO mice at P6, and in both naïve and EAE-affected adulthood (Table 3 and Fig 3B).

**Table 2:** Density of JV, PV, and Pa OPCs at P6^1^ and adulthood^2^.

	^3^Linear density		^4^Percentage of OPC subsets		
	JV	PV	JV	PV	Pa
P6 WT	0.89±0.22*	0.10±0.06	25.71±4.13*	4.54±1.22	70.38±3.17
P6 NG2KO	0.48±0.21	0.05±0.01	19.21±1.02	3.10±0.66	79.51±9.01
Naïve WT	0.47±0.13	0.02±0.01	10.09±10.82	0.78±0.51	81.25±7.89
Naïve NG2KO	0.31±0.08*	0.005±0.003	27.3±4.67	0.06±0.04	71.93±2.22
EAE WT 20dpi	**0.79±0.14**^#^	**0.23±0.11**^#^	**49.9±8.16**^#^	**7.84±3.62**^§^	**45.63±6.82**^#^
EAE WT 40dpi	0.59±0.13	0.05±0.07	**52.0±2.83**^#^	0.90±0.14	**46.01±2.83**^#^
EAE NG2KO 20dpi	0.50±0.14	0.05±0.04	29.05±15.22	4.44±4.07	68.56±14.62
EAE NG2KO 40dpi	0.61±0.16	0.03±0.07	27.35±13.37	2.34±2.70	73.56±10.59

^1^Unpaired t-test (P6 mice, n = 4) and

^2^Anova and Bonferroni tests (Adult mice, n = 5):

*p<0.05;

^**§**^p<0.01;

^**#**^p<0.001;

^3^OPC number/CVL (multiple of 100 μm of vessel length); mean ± SD;

^4^Number of each subset of OPCs over the total number of OPCs (PV + JV + Pa)*100; mean ± SD.

**Table 3:** Density of JV+PV doublet OPCs and Pa doublet OPCs at P6^1^ and adulthood^2^.

	^3^JV+PV linear density	^4^JV+PV volume density	^5^Pa volume density
P6 WT	**0.12±0.01**^#^	1.51±0.16*	0.36±0.51
P6 NG2KO	0.03±0.02	0.85±0.44	0.13±0.17
Naïve WT	0.04±0.02	0.75±0.52	0.18±0.08
Naïve NG2KO	0.02±0.02	0.49±0.27	0.12±0.04
EAE WT 20dpi	**0.11±0.03**^§^	**1.40±0.14**^#^	0.3±0.05
EAE WT 40dpi	0.02±0.01	0.41±0.03	0.33±0.07
EAE NG2KO 20dpi	0.03±0.04	0.36±0.18	0.18±0.1
EAE NG2KO 40dpi	0.06±0.06	0.39±0.21	0.27±0.17

^1^Unpaired t-test (P6 mice, n = 4 for each group) and

^2^Anova and Bonferroni tests (Adult mice, n = 5 for each group):

*p<0.05;

^**§**^p<0.01;

^**#**^p<0.001.

^3^JV+PV doublet OPC number/CVL (multiple of 100 μm of vessel length) (mean ± SD);

^4^JV+PV doublet OPC number/10^6^ μm^3^ of perivascular tissue (mean ± SD);

^5^Pa doublet OPC number/10^6^ μm^3^ of extravascular tissue (mean ± SD).

### NG2/CD13- and NG2/PDGFRβ-based morphometry detects activated pericytes in EAE

**Fig 4:**
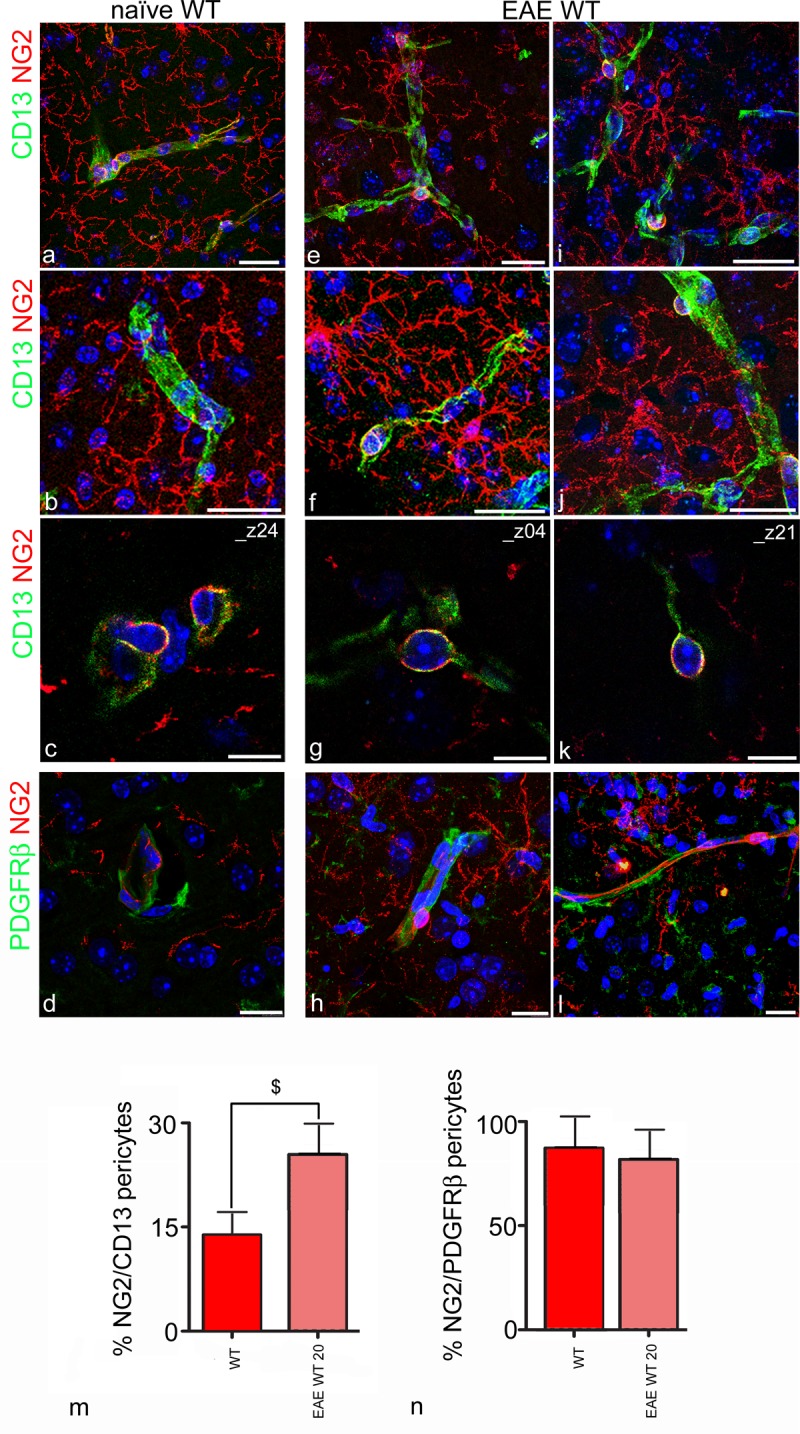
CD13/NG2-based morphometry detects activated pericytes in EAE. Representative confocal images of cerebral cortex sections double-immunolabelled for CD13 and NG2 (**a**-**c, e**-**k)** and for PDGFRβ and NG2 (**d**, **h**, **l**) to identify and quantify NG2^+^ activated pericytes in naïve (**a**-**d**) and EAE-affected (**e**-**l**) WT mice. A high number of hypertrophic, JV OPCs, which overexpress NG2 together with numerous CD13^+^/NG2^+^ activated pericytes are recognizable in cortical layers from 2 to 6 in EAE-affected (**e**-**j**), compared with naïve (**a**, **b**) WT mice. High magnification of single optical planes on ‘z’ axis (_z24 and _z04, _z21 in naïve and EAE-affected WT mice, respectively) localizes NG2 molecules on the plasma membrane around the cell nucleus, where the proteoglycan in part co-localizes with CD13 (**c**, **g**, **k**). PDGFRβ^+^/NG2^+^ pericytes are recognizible in cortical microvessels, NG2 molecules preferentially localize in cell bodies, whereas PDGFRβ in pericyte processes (**d**, **h**, **l**). **m** The bar chart shows a significant increase of activated pericytes in EAE-affected WT mice at 20 dpi, measured as the percentage of NG2^+^ pericytes over the total number of CD13^+^ pericytes (^$^*p* = 0.005; n = 4; unpaired Student’s t-test). **n** The percentageof NG2^+^ pericytes over the total number of PDGFRβ^+^ pericytes is similar in naïve and EAE-affected WT mice (*p* = 0.78; n = 4; unpaired Student’s t-test). EAE-affected WT mice (at 20 dpi: clinical score range = 2.0–3.5, mean number of counted PDGFRβ^+^ pericytes = 729 per mouse), EAE-affected NG2KO (at 20 dpi: clinical score range = 1.5–2.5, mean number of counted PDGFRβ^+^ pericytes = 459 per mouse). Nuclear counterstaining with TO-PRO-3. Scale bars, **a-l** 15 μm.

**Fig 5:**
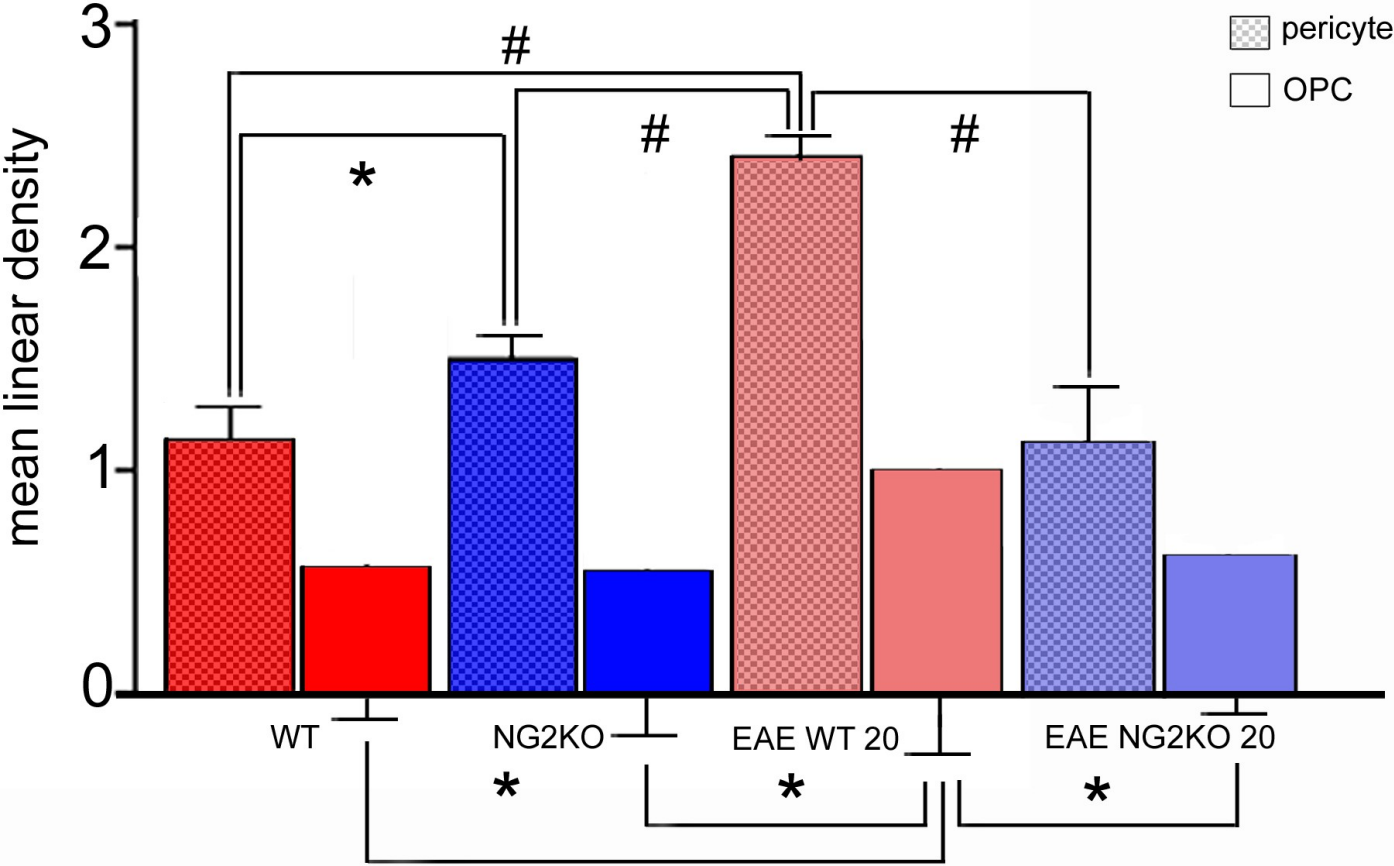
Comparison between the mean linear density of PDGFRβ^+^ pericytes and PDGFRα^+^ JV and PV OPCs. The PDGFRβ^+^ pericytes linear density (checkerboard pattern) and the PDGFRα^+^ JV and PV OPC linear density (smooth pattern), reported as number of identified cells on cumulative vessel length (multiple of 100 μm), are presented as mean ± SD. Direct comparison of linear density values calculated for pericytes and OPCs in WT, NG2KO, EAE-affected WT(20 dpi) and EAE-affected NG2KO (20 dpi) shows a consensual, significant increase at EAE WT (20 dpi). *p< 0.05, ^#^*p*< 0.0001. n = 5. EAE-affected WT mice (at 20 dpi: clinical score range = 2.0–3.5, mean number of counted PDGFRβ^+^ pericytes = 729 and PDGFRα^+^JV and PV OPCs = 312 per mouse), EAE-affectedNG2KO (at 20 dpi: clinical score range = 1.5–2.5, mean number of counted PDGFRβ^+^ pericytes = 459 and JV and PV OPCs = 136 per mouse).

Unlike CD45^+^/Iba1^+^ monocytes/macrophages, excluded by the NG2-based morphometry because only rarely seen to co-express NG2 (S1A–S1F Fig), immature pericytes express NG2 and PDGFRβ during development, in their re-activated states in pathological conditions [25–27], and as a baseline level in healthy adult mice (Fig 4A–4D and 4M). The rate of their re-activation in EAE-affected WT mice, measured on NG2/CD13 immunolabelled sections, demonstrated a significant increment of NG2^+^ pericytes in EAE-affected WT mice at 20 dpi (Fig 4E–4K and 4M). NG2/CD13 labelling, analysed on projection images (z-stacks) (Fig 4A, 4E, 4F, 4I and 4J) and single optical planes of the z axis (Fig 4C, 4G and 4K), immunolocalized the NG2 signal, in both naïve WT and EAE WT, to the pericyte cell body (Fig 4A, 4C, 4E–4K; see also S1 Movie, sequence of single optical planes from the z-stack in Fig 4I). In addition, the value of the ratio between the number of NG2^+^ and PDGFRβ^+^ pericytes, calculated for both naïve WT and EAE-affected WT mice, showed similar values, thus supporting the correspondence of the two markers for revealing activated pericytes (Fig 4D, 4H, 4L and 4N). The mutual contribution to the vessel wall of pericytes in their activated form (NG2^+^/PDGFRβ^+^) and of JV and PV OPCs (PDGFRα^+^) was then evaluated by measuring the linear density on PDGFRβ- and PDGFRα-stained sections (Fig 5). The direct comparison of density values calculated for vascular OPCs and activated pericytes showed a significant increase in both cell types in EAE-affected WT mice at 20 dpi compared to naïve WT, naïve NG2KO, and EAE-affected NG2KO mice (Fig 5). A significant increase in PDGFRβ+ pericytes in naïve NG2KO compared to naïve WT mice was also observed (Fig 5).

### Endothelial tight junctions and barrier tightness are maintained in EAE-affected NG2KOmice

**Fig 6:**
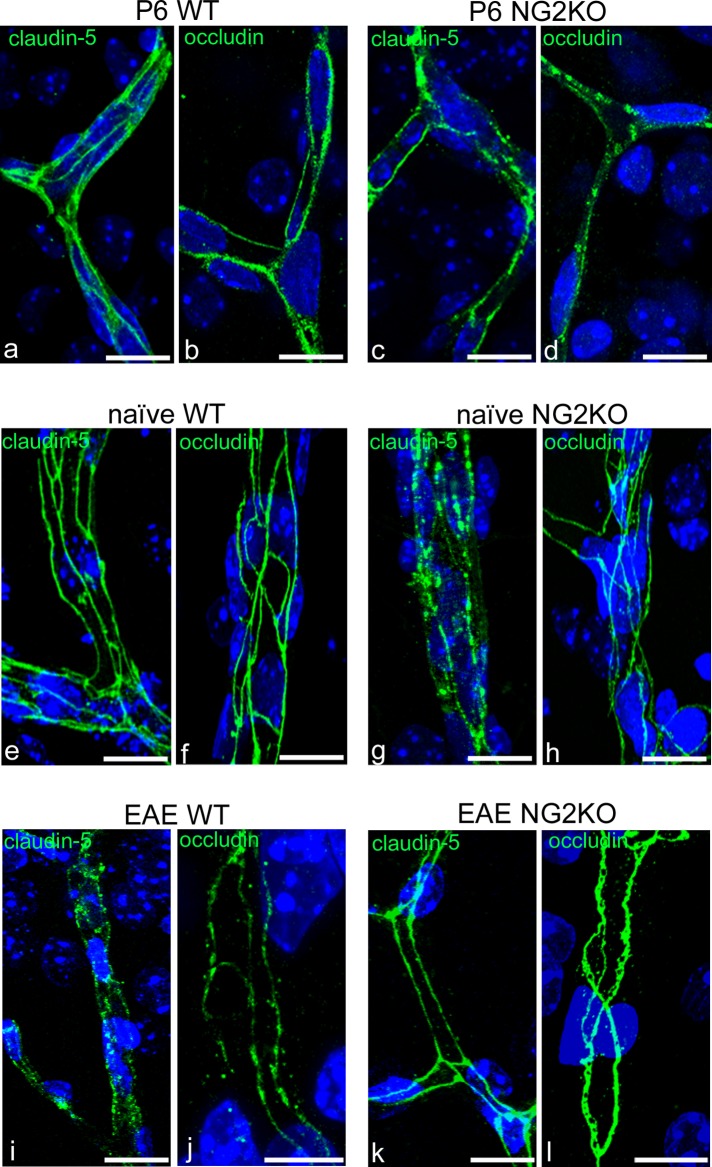
Endothelial tight junctions are maintained in EAE-affected NG2KO mice. **a**-**l** Representative confocal images of cerebral cortex sections immunolabelled for claudin-5 and occludin. **a**, **b**, **e**, **f** The cerebral cortex microvessels show continuous staining patterns for both claudin-5 and occludin in P6 WT and adult naïve WT mice. **c**, **d**, **g**, **h** Claudin-5 and occludin appear irregularly organized in P6 NG2KO mice, with these modified patterns persisting in adult, naïve NG2KO mice; note the typical chain-like claudin-5 pattern in (**g**). **i**, **j** In the cortex microvessels of EAE WT mice, claudin-5 and occludin are lost along junctional tracts, whereas in EAE-affected NG2KO mice TJs proteins staining appears reinforced (**k**, **l**) and occludin acquires a ‘perforated ribbon-like’ configuration, with full and empty tracts. Nuclear counterstaining with TO-PRO-3. Scale bars, 10 μm.

**Fig 7:**
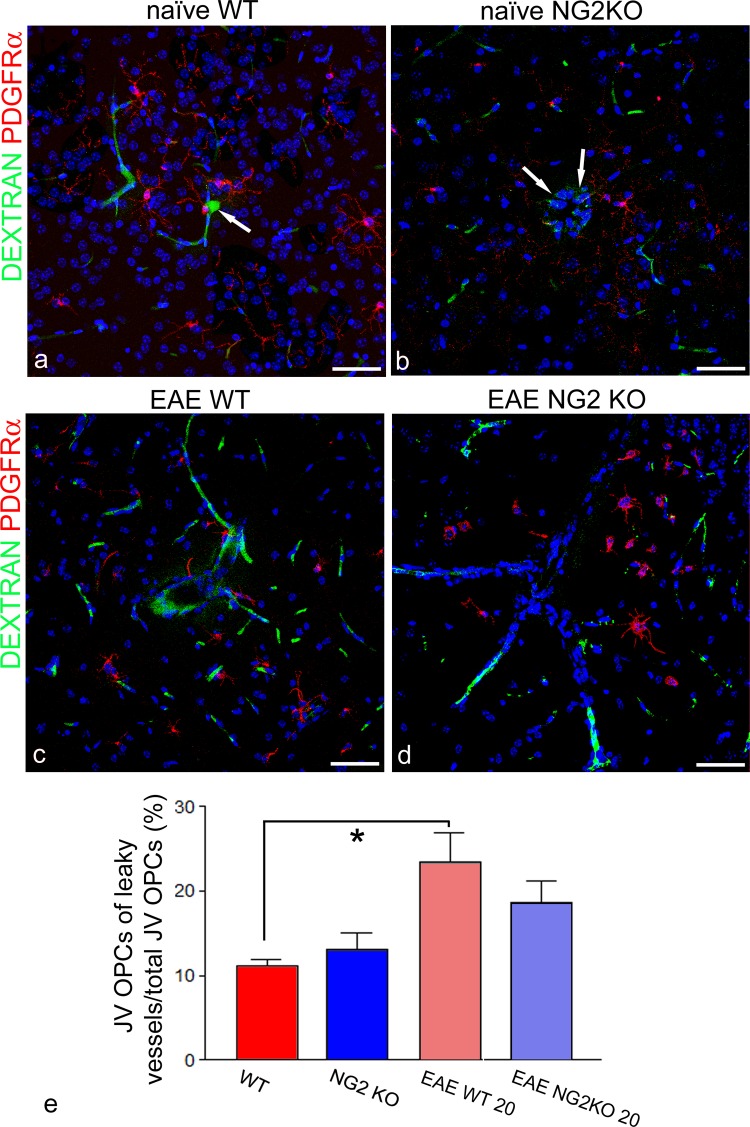
Barrier tightness is maintained in EAE-affected NG2KO mice. **a**-**d** Representative confocal images from mice injected with FITC-Dextran 70 kDa for BBB permeability assessment, then immunolabelled for PDGFRα to show vessel/OPC association. **a**, **b** The exogenous tracer stains the vessel lumen in BBB cerebrocortical microvessels of both naïve WT and naïve NG2KO mice: note in (**a**) a transversely cut microvessel with FITC-dextran in the vessel lumen (arrow) and in (**b**) a minimal leakage around a small venule (arrows). **c**, **d** The deep cerebral cortex microvessels (at the boundary with the subcortical white matter) of EAE-affected WT mice appear permeable to the exogenous tracer that forms a fluorescent halo in the surrounding neuropil (**c**), while little signs of increased permeability are shown by cortex microvessels in EAE-affected NG2KO mice (**d**). **E** The bar chart shows the percentage of JV OPCs associated with leaky microvessels, a value that is significantly increased in EAE-affected WT mice (20 dpi) compared with naïve WT mice. No significant differences are observed between naïve NG2KO and EAE NG2KO mice (mean ± SD; n = 5;*p <0.*05*). FITC-dextran injected EAE WT mice (20 dpi clinical score range: 1.5–2.5, mean number of counted JV OPCs contacting leaky vessel: 14 per mouse), FITC-dextran injected EAE NG2KO (20 dpi clinical score range: 1.5–2.5, mean number of counted JV OPCs contacting leaky vessel: 5 per mouse). Nuclear counterstaining with TO-PRO-3. Scale bars, 50 μm.

The possible role of NG2 on the BBB function was investigated through immunolocalization of junctional BBB proteins, claudin-5 and occludin, in cerebral cortex microvessels of WT and NG2KO P6 mice, and adult naïve and EAE-affected mice, both WT and NG2KO (Figs 6A–6L and S2). In P6 WT mice, both claudin-5and occludin immunostaining revealed an adult-like, regular, linear and continuous staining pattern, with some areas of occludin punctate staining (Figs 6A and 4B). In P6 NG2KO, TJ staining for both claudin-5 and occludin showed thinner linear tracts and smaller punctate areas (Figs 6C and 6D and S3A and S3A’). At this early developmental stage, while the evidence of adult-like TJs indicated the presence of stable microvessels, the presence of vessel sprouts implied an ongoing process of cortex vascularization, which in rodent brain proceeds until 2–3 weeks after birth, with a peak in the first postnatal week [28]. Accordingly, in P6 WT mice, there was marked, diffuse claudin-5 staining of vessel sprouts (S3A–S3D Fig), which, in contrast, were hardly detectable in P6 NG2KO mice (S3E and S3F Fig), and appeared reduced in number (sprouting points *per* CVL = 52±19.35% less in P6 NG2KO *vs* P6 WT mice; *p* = 0.0238; n = 4). In adult naïve WT mice, claudin-5 and occludin displayed a typical, linear and regular junctional pattern (Figs 6E and 6F and S2B and S2B’), different from those revealed in adult naïve NG2KO mice that were characterized by a claudin-5 chain-like staining formed by fluorescent alternate large spots and thin tracts (Figs 6G and S2C and S2C’) and by occludin ribbons of irregular widths (Fig 6H). In EAE-affected WT mice, claudin-5 and occludin revealed a punctate and interrupted junctional staining pattern and completely unstained vessel tracts (Fig 6I and 6J). What looked like a dismantled junctional architecture in EAE WT mice was not recognizable in EAE NG2KO mice, which instead, showed wide uninterrupted, linear patterns (Fig 6K and 6L). A detailed observation of the junctional areas, immunostained for occludin, revealed a ‘perforated ribbon’ aspect (Fig 6L). The experiments carried out with FITC-dextran were consistent with these observations (Fig 7A–7D). In fact, FITC-dextran appeared restricted to the lumen of cortex microvessels in naïve WT and NG2KO mice (Fig 7A and 7B), whereas it formed a fluorescent perivascular halo in EAE-affected WT mice (20 dpi) that was absent in EAENG2KO mice (20 dpi) (Fig 7C and 7D) [11]. The highest number of leaky microvessels was measured in the cerebral cortex of EAE-affected WT mice (Table 4), which also revealed, by combined FITC-dextran/PDGFRα-based morphometry (Fig 7C), the highest density *per* CVL of vascular OPCs(JV + PV) associated with leaky vessels (Table 4) and the highest percentage of JV OPCs *per* leaky vessels over the total number of JV OPCs (Fig 7E). No significant difference was observed between naïve NG2KO and EAE-affected NG2KO mice (Fig 7E).

**Table 4:** JV+PV OPCs associated with non-leaky *vs* leaky microvessels.

	^1^Number of leaky/total vessels	^2^Density of OPCs *per* non–leaky vessels	^2^Density of OPCs *per* leaky vessels
Naïve WT	8/449	0.46±0.36	0.81±0.58
Naïve NG2KO	5/504	0.33±0.07	0.35±0.36
EAE WT 20dpi	26/478*	0.36±0.1	**0.78±0.15**^#^
EAE NG2KO 20dpi	16/428	0.15±0.17	0.31±0.29

^1^Anova and Bonferroni tests:

*p<0.05;

^2^Unpaired t-test:

^**#**^p<0.001 (mean ± SD).

### NG2KO mice show reduced laminin and collagens VI and IV in the vessel basal lamina

**Fig 8:**
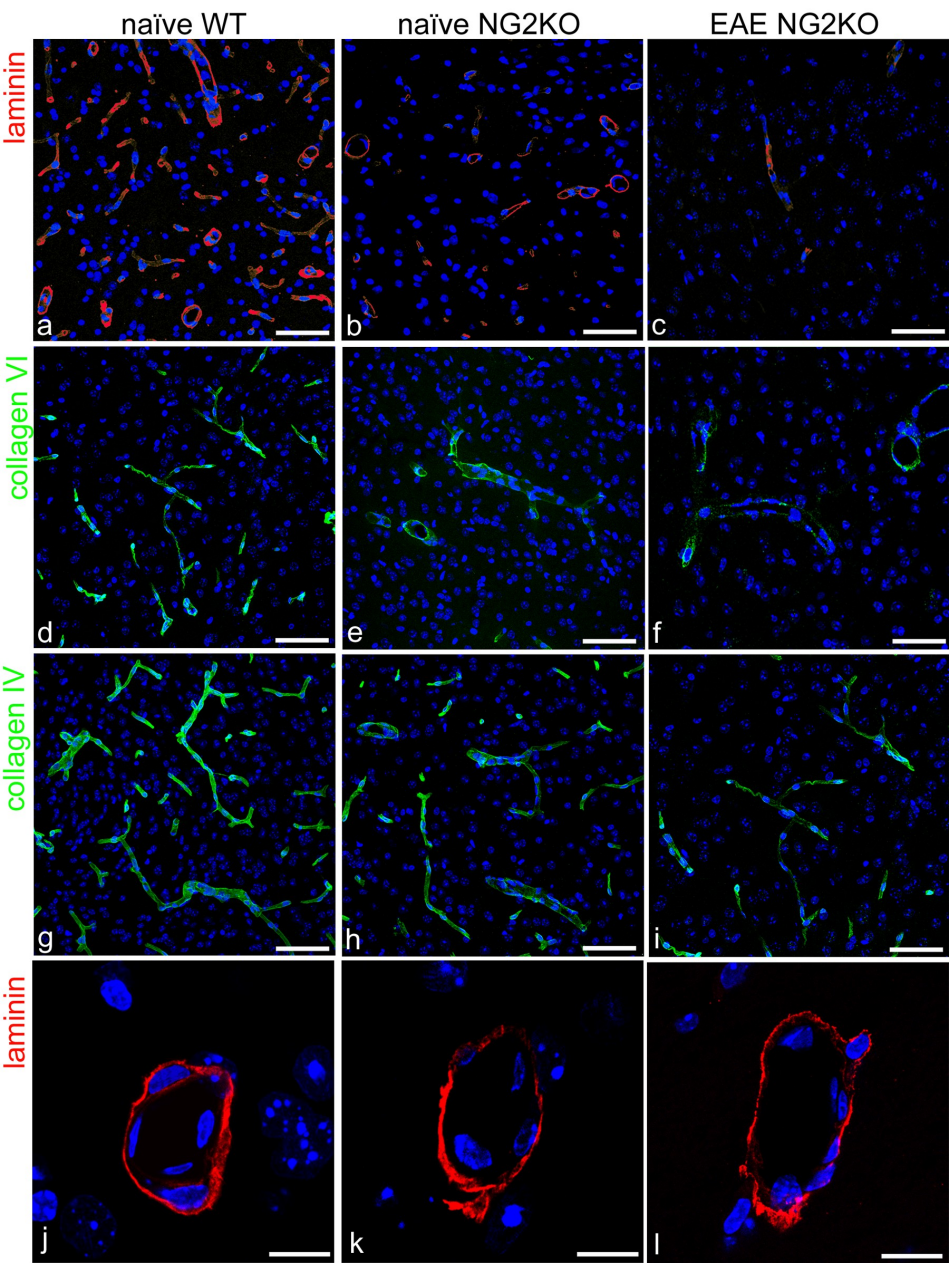
NG2KO mice show reduced laminin and collagens VI and IV in the vessel basal lamina. **a**-**l** Representative confocal images of cerebral cortex sections immunolabelled for laminin, collagen VI and IV. Compared with naïve WT mice (**a**, **d**, **g**) the staining of the examined VBL molecular components appears reduced in both naïve and EAE-affected NG2KO mice (**b**, **c**, **e**, **f**, **h**, **i**). **j**-**l** The differences in laminin immunostaining between naïve WT and naïve or EAE-affected NG2KO mice are better seen at a higher magnification, which also served for confocal morphometry. Nuclear counterstaining with TO-PRO-3. Scale bars, **a-i** 30 μm and **j-l** 10 μm.

The analysis of the cerebral cortex microvessels in adult WT and NG2KO mice was extended to vessel basal lamina (VBL) molecules, which are well known NG2 ligands, laminin and collagen VI, or are tightly linked to a ligand, such as occurs with the collagen VI/collagen IV molecular binomial. When compared with naïve WT, both naive NG2KO and EAE-affected NG2KO mice showed markedly reduced staining for laminin and collagen VI, as well as collagen IV (Fig 8A–8I; see also S3E and S3F Fig). The laminin reduction was quantified by measuring the percentage of laminin-positive pixels on the total pixel number (‘area fraction’ expressed as mean ± SD) and evaluating the laminin VBL thickness (Fig 8J–8L). Compared with adult naïve WT, the laminin area fraction of naïve NG2KO was reduced by 39.5±5.6% (p<0.001; n = 5) and laminin thickness by 44.7±2.8% (p<0.001; n = 5).

### VEGF-A and VEGFR2 are the candidate pathways in NVU OPC recruitment

**Fig 9:**
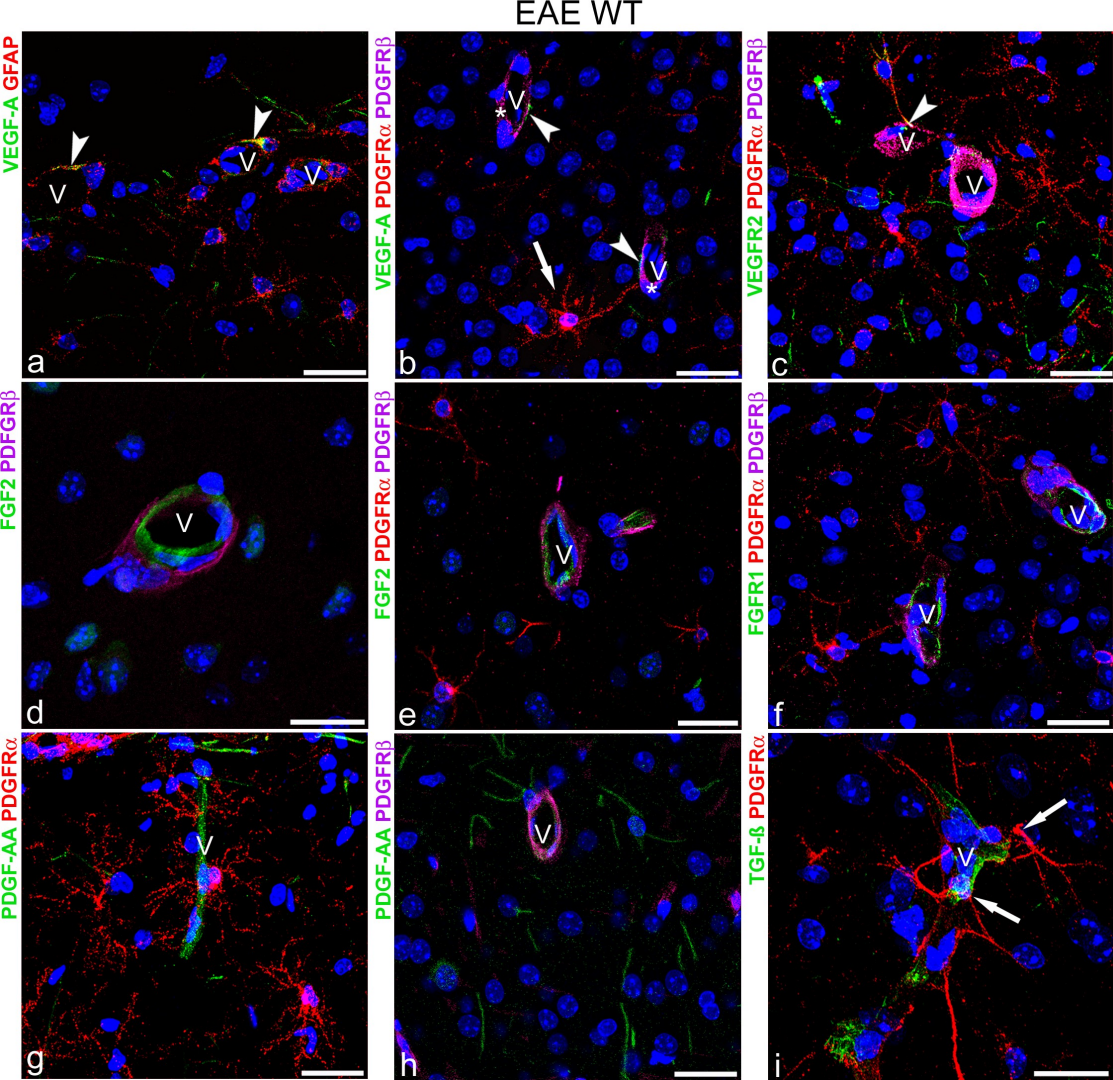
VEGF-A and VEGFR2 as candidate pathway in NVU OPC recruitment. **a**-**i** Representative confocal images of cerebral cortex sections from EAE-affected WT mice, immunolabelled for growth factors and their receptors: VEGF-A and its receptor VEGFR2 (**a**-**c**), FGF2 and its receptor FGFR1 (**d-f**), PDGF-AA/PDGFRα (**g, h**) and TGFβ (**i**), in combination with GFAP, PDGFRα, and PDGFRβ, as cell-specific markers. **a** VEGF-A co-localizes with GFAP in astrocyte endfeet (arrowheads) on vessel wall (V). **b** A typical PDGFRα^+^ JV OPC (arrow) and PDGFRβ^+^pericytes (asterisks) do not show VEGF-A reactivity; note VEGF-A^+^ astrocyte-like endfeet (arrowheads). **c** VEGFR2 co-localize with PDGFRα^+^on a JV OPC (arrowhead). **d**, **e** FGF2 is localized on endothelial cells and stains neither PDGFRβ^+^ pericytes (**d**) nor PDGFRα^+^ OPCs (**e**). **f** FGFR1 is localized on endothelial cells but not on pericytes or OPCs. **g**, **h** PDGF-AA stains endothelial cells of cortex microvessels surrounded by PDGFRα^+^ JV and PV OPCs (**g**) and by PDGFRβ^+^ pericytes (**h**). **i** The endothelial cells of a cortex microvessel contacted by PDGFRα^+^ processes (arrows) are immunoreactive for TGFβ. Nuclear counterstaining with TO-PRO-3. V, vessels. Scale bars, **a-d** and **f-i** 30 μm, **e** 15μm.

**Fig 10:**
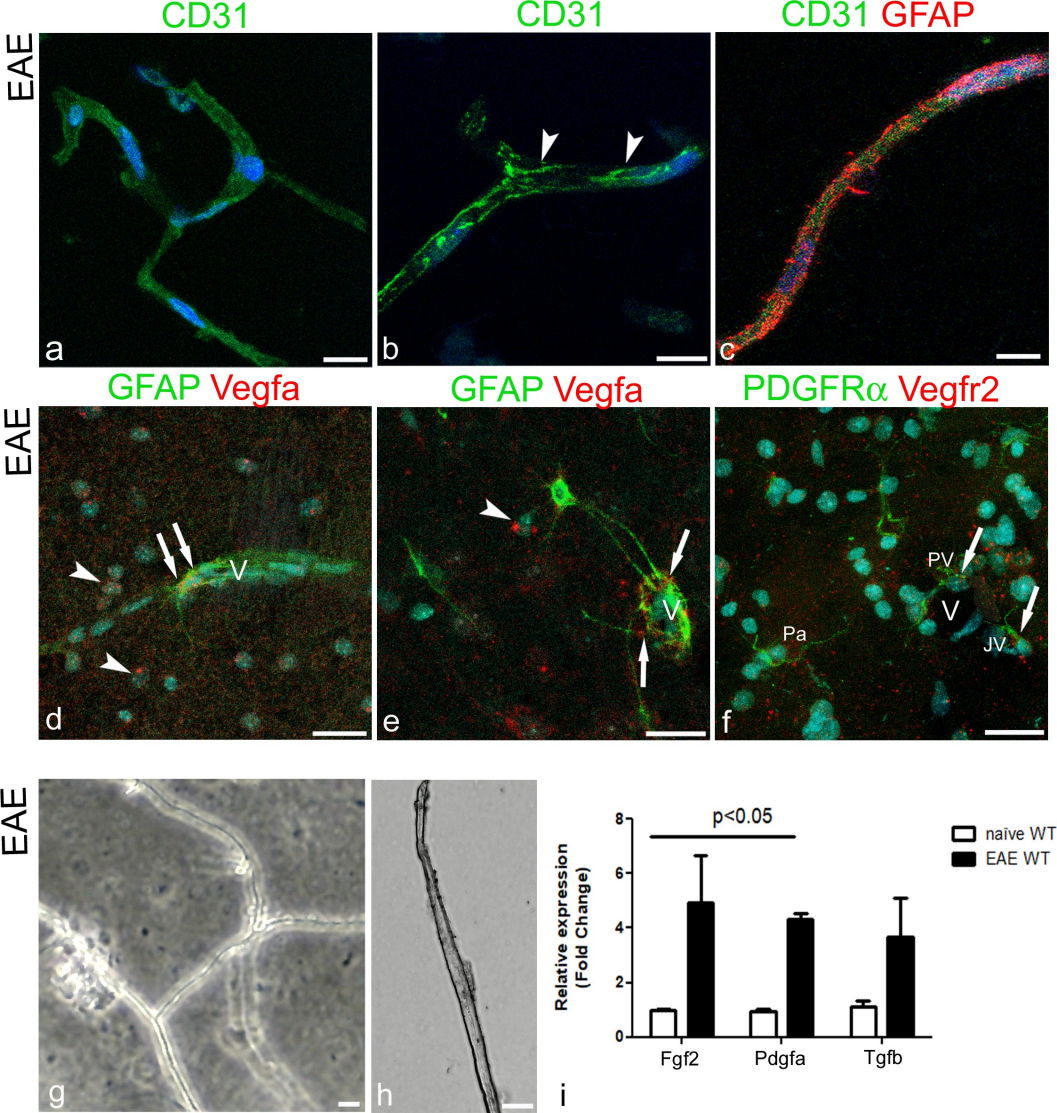
Expression of growth factors on isolated brain microvessels and cerebral cortex sections. **a-c** Representative confocal images of cerebral cortex isolated microvessels from EAE-affected mice, immunolabelled for CD31 (**a, b**) and for CD31/GFAP (**c**). **a, b** CD31 identifies the endothelial cells and appears localized at the junctional endothelial contacts (**b,** arrowheads). **c** GFAP^+^ astrocyte endfeet are recognizable in the close vicinity of the CD31-stained vessel wall. **d-f** Representative confocal images of cerebral cortex of EAE-affected WT mice processed for the detection of GFAP antibody/Vegfa mRNA and PDGFRβ antibody/Vegfr2 mRNA by dual RNAscope IHC/ISH. **d** GFAP^+^ perivascular astrocyte close to the vessel wall (V), and (**e**) perivascular astrocyte processes on a microvessel (V) show red fluorescent puncta corresponding to Vegfa ISH signal (arrows); note fluorescent puncta also in nuclei of neuron-like cells (arrowheads); **f** PDFGRα^+^ perivascular (PV), juxtavascular (JV) and parenchymal (Pa) OPCs show Vegfr2 ISH signal in the cell body (arrows). **g, h** Phase contrast microscopy images of the pelleted microvessels. **i** Real-time PCR analysis of mRNAs expression of Fgf2, Pdgfa and Tgfb in cerebral cortex of naïve and EAE-affected WT mice. The analysis by real-time PCR shows a significant increase in Fgf2 and Pdgfa levels in the isolated vessels of cerebral cortex from EAE-affected compared with WT mice. Instead, Tgfb expression shows no statistically significant difference. Nuclear counterstaining with TO-PRO-3 (**a-c**) and Sytox Green (**d-f**). Scale bars, **a**-**f** 25 μm and **g**, **h** 20 μm.

Several canonical signalling pathways, which are known to work within the NVU and could be involved in the described increase of vascular OPCs resulting from proliferation and/or induced migration and recruitment, were immunolocalized on cerebral cortex sections from EAE-affected WT mice. Double and multiple immunolabellings were carried out with growth factors, VEGF-A (vascular endothelial growth factor A), PDGF-AA (platelet derived growth factor-AA), FGF2 (fibroblast growth factor-2), and TGF-β (transforming growth factor-β) and their receptors (Fig 9A–9I). By double staining with VEGF-A and glial fibrillary acidic protein (GFAP), a marker of astrocytes, VEGF-A was localized at the endfeet of perivascular astrocytes (Fig 9A), while on VEGF-A/PDGFRα/PDGFRβ labelled sections, PDGFRα^+^ vascular OPCs and PDGFRβ^+^ pericytes were VEGF-A^-^ (Fig 9B). When immunostaining for PDGFRα/PDGFRβ were combined with that forVEGFR2 (vascular endothelial growth factor receptor 2), VEGFR2 was detected on vascular PDGFRα^+^ OPC processes (Fig 9C). FGF2 was immunolocalized on the endothelial lining and did not co-localize with PDGFRα and PDGFRβ on OPCs and pericytes, respectively (Fig 9D and 9E). Its receptor FGFR1 (fibroblast growth factor receptor 1) stained endothelial cells and also co-localized with PDGFRβ but not with PDGFRα (Fig 9F). PDGF-AA/PDGFRα, PDGF-AA/PDGFRβ double immunostainings showed PDGF-AA-positive endothelial cells (Fig 9G and 9H) and astrocyte-like processes (Fig 9H), while TGF-β was revealed on the endothelium-pericyte layer, surrounded by PDGFRα^+^ OPC processes (Fig 9I). On the same experimental group of mice, transcript expression of the immunolocalized growth factors (Fgf2, Pdgfa, and Tgfb) were analysed by real time-PCR on isolated cerebral cortex vessels and by dual RNAscope-immunohistochemistry/in situ hybridization (IHC/ISH) on cerebral cortex sections (Vegfa/Vegfr2). The protocol used for isolation of microvessels from fixed cerebral cortices was developed by optimizing the given Dissociation Kit (see M&M section), and it was checked for consistency by phase contrast microscopy and immunohistochemistry for specific cell markers (Fig 10A–10C, 10G and 10H). Single CD31 and combined CD31/GFAP stainings confirmed the presence of microvascular fragments (containing endothelial cells, perivascular astrocyte endfeet, and possibly pericytes; Fig 10A–10C). Real time-PCR for Fgf2, Pdgfa, and Tgfb mRNA of the filtered pellets that contained the isolated microvessels (Fig 10G and 10H) show that these transcripts were all increased in EAE-affected mice compared to naïve WT mice (Fig 10I). In addition, by dual RNAscope technique, Vegfa and Vegfr2 transcripts were revealed on cerebral cortex sections, together with the cell-specific markers GFAP, for astrocytes, and PDGFRα, for OPCs at protein level (Fig 10D–10F). On GFAP-IHC/Vegfa-ISH stained sections, GFAP^+^ perivascular astrocyte showed red fluorescent puncta corresponding to Vegfa RNAscope signal (Fig 10D and 10E); on these sections, the fluorescent Vegfa probe was also seen on nuclei of neuron-like cells (Fig 10D and 10E). On PDGFRα-IHC/Vegfr2-ISH stained sections, a number of vascular OPCs and Pa OPCs were revealed by anti-PDGFRα antibody and were marked by the fluorescent probe for Vegfr2 mRNA (Fig 10F).

## Discussion

As demonstrated by *in vivo* time-lapse imaging in mouse brain, OPCs are highly motile cells that actively migrate and extend processes to survey the homeostasis of their own territory in both physiological and pathological conditions [29]. The basic idea of this study is that such a dynamic type of glial cells may contribute to nervous tissue development and/or response to injury [30–32] through their recruitment and active participation to the NVU, and that part of the biological functions related to OPC/vessel interactions may be mediated by NG2 proteoglycan. Interestingly, in the CNS, two cell types primarily express NG2: OPCs, which express NG2 during development (proper OPCs) and continue to express the proteoglycan in adulthood (NG2-glia) [33], and pericytes, which upregulate NG2 expression according to their developmental (‘immature’ pericytes) and pathological (re-activated pericytes) status, and downregulate the proteoglycan in adult brain [25, 34].

The quantitative data obtained in this study by immunoconfocal morphometry demonstrated that OPCs are integral components of the cerebral cortex NVU and that their contribution varies according to physiological and pathological conditions. The baseline level of NVU's OPCs detected in adult, naïve WT mice paralleled the revealed low percentage of NG2^+^ pericytes, both possibly reflecting the moderate plasticity displayed by cerebral cortex microvessels throughout adult life [35]. However, vascular OPCs in WT mice were shown to fluctuate from a peak observed at P6, which wanes in adulthood, to a new increase during EAE, a time course which mirrored that of the immature/activated pericytes, and which was not the case for both OPCs and pericytes in NG2KO mice. In addition, the analysis of vascular OPCs, combined with TJ protein localization at early stages of postnatal development and in naïve and EAE-affected WT and NG2KO mice, together with the synchronized activation (NG2 re-expression) of pericytes, indicates NG2 proteoglycan as one of the molecules involved in OPC/pericyte/BBB-endothelial cell interaction. Literature data suggest that OPCs and pericytes share regulative molecules, which possibly result in redundant signalling pathways. In fact, as already demonstrated for pericytes [36], specific genetic ablation of OPCs leads to severely impaired brain vascularization [13], and while pericytes, in co-culture with endothelial cells, secrete TGF-β promoting BBB formation *via* TGF-βR2-ALK5-Smad2/3 pathway [37], OPCs, in co-culture with endothelial cells are able to support BBB integrity by secreting TGF-β and activating TGF-βR-MEK/ERK signalling pathway [38]. Interestingly, in double monolayer culture models, NG2 knockdown in pericytes reduces barrier function [17], whereas in white matter injury, NG2-expressing OPCs were demonstrated to degrade TJ-associated ZO-1 [39]. Our previous findings concerning TJ response and BBB function in the same model of EAE, where the cerebral cortex is affected, showed abnormal TJs and BBB leakage in diseased cortices [10], and revealed that endothelial TJs perform better in EAE NG2KO mice, compared with EAE WT mice [11]. In the present study, NG2-null mice at P6 show a modified arrangement of the junctional strands that persist in adult naïve NG2KO mice. While TJ and BBB alterations observed in EAE WT mice coincide with the highest percentage of vessel-associated OPCs, in EAE NG2KO mice, which lack vessel OPC increase, TJ staining appears restored to a continuous and possibly improved pattern. These apparently contradictory results support the hypothesis of differential NG2 effects in developmental *vs* adulthood and in pathological conditions, according to the existence of different NG2 isoforms (glycoforms?) [40] and/or NG2 distinct phosphorylated forms, which may modify NG2 cell/molecular interactions [41]. Moreover, it has been recently shown that cleavage, by α- and γ-secretases, leads to four NG2 fragments that have been associated with different biological functions in the CNS [42]. The atypical holed ribbon-like profile revealed by occludin in EAE NG2KO mice, further supports this idea, for which we propose the following interpretation: although upon NG2 ablation, the EAE NVU microenvironment may mimic the adult healthy-like condition, multiple inflammatory cues persist in the cerebral cortex of EAE NG2KO mice, and under their effect endothelial cells assume a morphological complexity with a convoluted profile [27, 43], increased junctional, lateral contacts, overlapping cell borders and interdigitated finger-like processes [44]. Knowledge of the biological activity exerted by NG2 in the vascular system, translated to vascular OPCs, can help to understand the described effect of NG2 expression and of its ablation on endothelial TJ regulation. NG2 displays limited capability for independent signal transduction, but in addition to its role as regulator of cell surface domains and growth factor activities [45, 46], NG2 may exert a direct effect, working in a *trans* mode by activating β1-integrin signalling on the closely apposed endothelial cells [16, 17, 47]. β1-integrin-mediated anchoring of endothelial cells to VBL molecules is involved in intercellular signalling, which stabilizes claudin-5 localization in TJ and BBB integrity [48, 49]. It has been emphasized that BBB properties are not simply ‘switched on’ in developing brain endothelium, but, rather, continuous signals from cellular and non-cellular components of the NVU are needed to maintain TJ integrity [50–51]. As an example, the differential β1-integrin-mediated effects on nascent endothelium *vs* a fully differentiated BBB may lead to distinct downstream signalling events [51]. Moreover, accessory molecules involved in NG2/β1-integrin *trans* activity can differentially contribute to TJ regulation according to their broad range of activities in physiological, as well as pathological, conditions. One of these molecules, galectin-3, characterized by combined self-association and carbohydrate recognition domains, may form pentamers that determine integrin clustering [52]. GST-fusion protein pull-down assays demonstrated a direct interaction between the NG2 D3 ectodomain (the membrane proximal segment) and galectin-3, with formation of NG2-galectin-3-α3β1 integrin tri-molecular complexes at the pericyte-endothelial interface [16]. Finally, multimerization of cell surface complexes serves as a mechanism for amplifying integrin-mediated transmembrane signaling [53]. The possibility that this mechanism can be applied to OPC/endothelial cell interaction, is sustained by immunoelectron microscopy studies that described direct adhesion of OPCs to cerebral endothelium *via* vessel basal lamina [38]. Moreover, binding of NG2 to laminin and collagen VI [48, 54–57] and, indirectly, to collagen IV [58], assists the retention at the cell surface and their dynamic assembly [56]. In this context, the expected significant reduction in laminin and collagens VI and IV observed in NG2KO mice [58], may participate in determining TJ abnormalities [59, 60] described in P6 and naïve NG2KO mice. The analysis of the ligand/receptor systems possibly involved in vascular OPC proliferation and migration/recruitment in EAE-affected WT mice, points out to VEGF-A and PDGF-AA as the primarily involved factors. In fact, although endothelial-derived FGF2 has been described as promoting OPC proliferation *in vitro* [61], mice lacking both FGFR1 and FGFR2 exhibit normal OPC proliferation and differentiation [62]. The detected expression of both FGF2 and FGFR1 on the endothelial cells and the increase of the Fgf2 transcript in cortex microvessels isolated from EAE-affected WT mice seems to confirm the importance of FGF2/FGFR1 signalling in affecting several endothelial cell functions through an autocrine mode of action [63, 64], rather than their involvement in OPC proliferation and/or migration. TGF-β, which is known to contribute to survival of endothelial cells and BBB-microvessel function [65, 66], can work as an indirect mitogen for vascular OPCs. In fact, co-stimulation with pericyte-derived TGF-β and astrocyte-derived PDGF-AA [67, 68], selectively enhances PDGFRα synthesis and surface expression, thereby amplifying the proliferative response of OPCs to PDGF-AA [38, 69]. Vascular endothelial growth factor A (VEGF-A) is best known for its essential roles in blood vessel growth in normal and pathological conditions. However, evidence has emerged that VEGF-A also exerts direct activities on neurons during EAE [70] and that reactive astrocytes express VEGF-A in multiple sclerosis [71]. These data together with the documented expression of VEGFR2 by OPCs [72], confirm the results obtained by IHC and RNAscope IHC/ISH on the expression of Vegfa by perivascular astrocytes and of Vegfr2 by vascular OPCs, suggesting a direct cross-talk between these cells within the NVU during EAE.

Overall, this study support the idea that similarly to 'neurovascular niches', where endothelial cells produce molecular signals that sustain neuronal precursors, 'oligovascular niches' also exists, where endothelial cells provide trophic factors to sustain oligodendrocyte precursors (OPCs/NG2-glia) [61] and where mutual and multiple interactions occur which involve the NVU cell components, including vascular OPCs. In fact, the described dynamic vascular setting of OPCs in both physiological and perturbed NVU conditions, allows to propose these cells as a novel NVU component. In addition, the shared expression of NG2 with immature/activated pericytes suggests that OPCs may exert part of their biological functions within the NVU possibly through NG2 *trans* activity and/or by interactions of NG2 with VBL molecules, and/or by the regulative activity of the proteoglycan on growth factor availability/accessibility at the cell membrane [73].

## Supporting information

S1 Fig Cerebral cortex monocytes/macrophages and microglia rarely express NG2 proteoglycan. **a-f** Representative confocal images of brain sections from EAE-affected WT mice, double immunolabelled for NG2/CD45 (**a-c**) and NG2/Iba1 (**d-f**) showing that, among monocytes/macrophages of inflammatory infiltrates, rare co-localizations of NG2 and CD45 or Iba1 are appreciable in EAE mice and these NG2^+^monocytes/macrophages are ovoid cells devoid of processes (arrows), morphologically different from OPCs and pericytes. Scale bars, 25 μm.
(TIF)

S2 Fig The staining pattern of endothelial tight junctions is modified in NG2KO. **a-c, a’-c’** Comparison of claudin-5 staining patterns between NG2KO mice, both at P6 (**a**) and in adulthood (**c**), with adult, naïve WT mice (**b**). **a’**, **b’**, **c’** The binary black and white format of the same images shown in (**a**, **b**, **c)** better demonstrates the differences between claudin-5 junctional patterns. Nuclear counterstaining with TO-PRO-3 in (**a**-**c**). Scale bars, 10 μm.
(TIF)

S3 Fig Claudin-5 expression also reveals vessel sprouts. **a**-**f** Representative confocal images of vessel sprouts double-immunolabelled for claudin-5 and collagen IV. **a**-**d** In P6 WT, endothelial stalk and tip cells show a strong, diffuse claudin-5 staining, which also reveals typical filopodial extensions. **e**, **f** In P6 NG2KO mice, sprouting endothelial tip cells show a punctate claudin-5 staining; note the reduced collagen IV staining between WT (**a**-**d**) and NG2KO sprouts (**e**, **f**). Nuclear counterstaining with TO-PRO-3. Scale bars, **a-f** 10 μm.
(TIF)

S1 Movie Representative movie file from which image in Fig 4B is derived, showing two CD13^+^ (green)/NG2^+^ (red), activated pericytes of a cerebral cortex microvessel of a naïve WT mouse. This imaging segment of xy single optical planes through the 'z' axis shows that NG2 is mainly restricted to plasma membrane of pericyte bodies, whereas CD13 localizes on both pericyte bodies and processes.
(AVI)

S1 Excel file Raw, numeric data of morphometric analyses of OPCs, pericytes, blood vessels, sprouting points, and laminin utilized for calculating statistical significance of differences among the experimental groups. (XLSX)
