# Supplementary figures and images for: Defining the role of NG2-expressing cells in experimental models of multiple sclerosis. A biofunctional analysis of the neurovascular unit in wild type and NG2 null mice
Source: PLoS One. 2019 Mar 14;14(3):e0213508. doi: 10.1371/journal.pone.0213508 (PMC6417733; doi:10.1371/journal.pone.0213508)

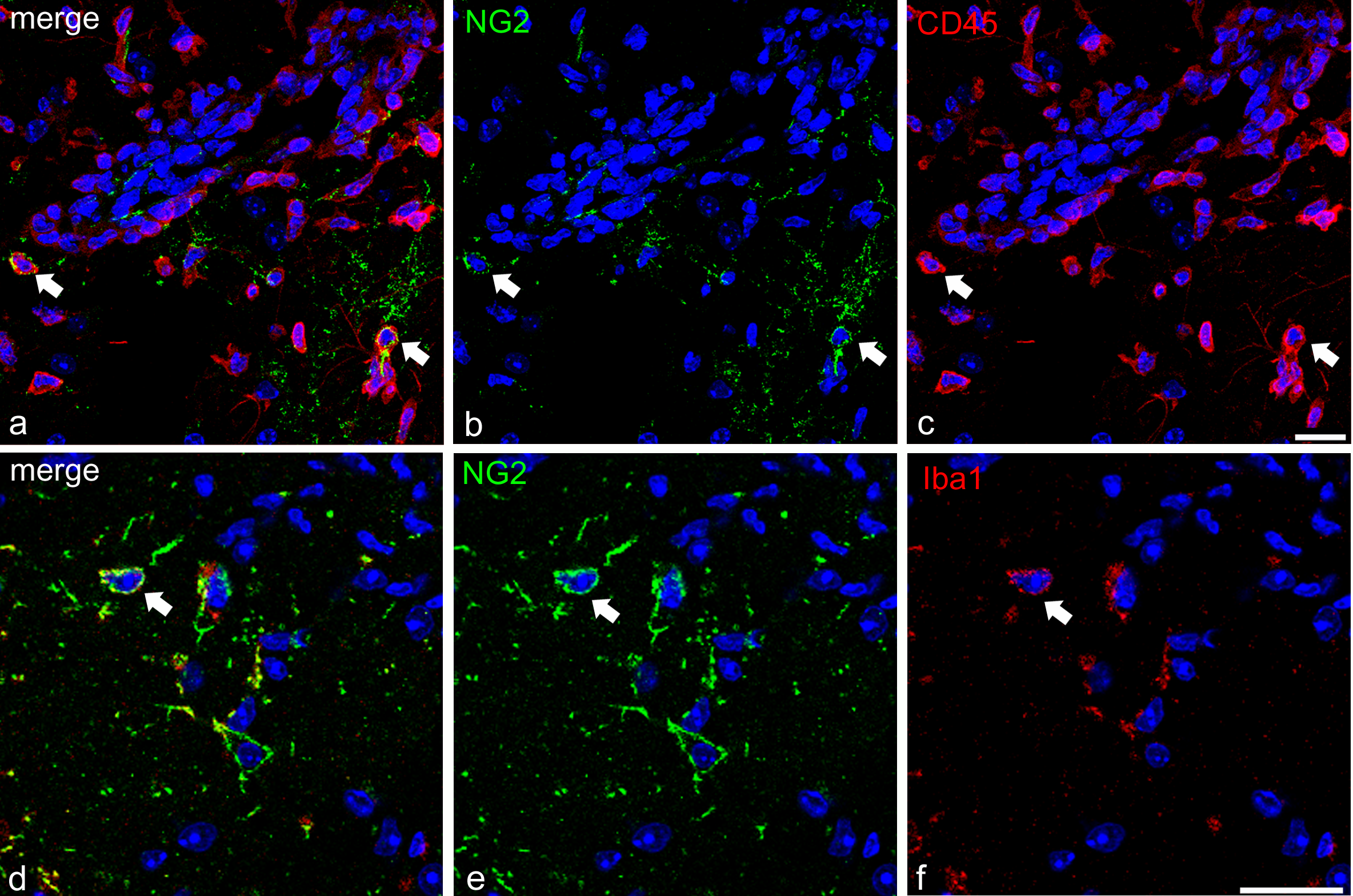

Supplement: S1 Fig — a-f Representative confocal images of brain sections from EAE-affected WT mice, double immunolabelled for NG2/CD45 (a-c) and NG2/Iba1 (d-f) showing that, among monocytes/macrophages of inflammatory infiltrates, rare co-localizations of NG2 and CD45 or Iba1 are appreciable in EAE mice and these NG2+monocytes/macrophages are ovoid cells devoid of processes (arrows), morphologically different from OPCs and pericytes. Scale bars, 25 μm. (TIF) [file pone.0213508.s001.tif]

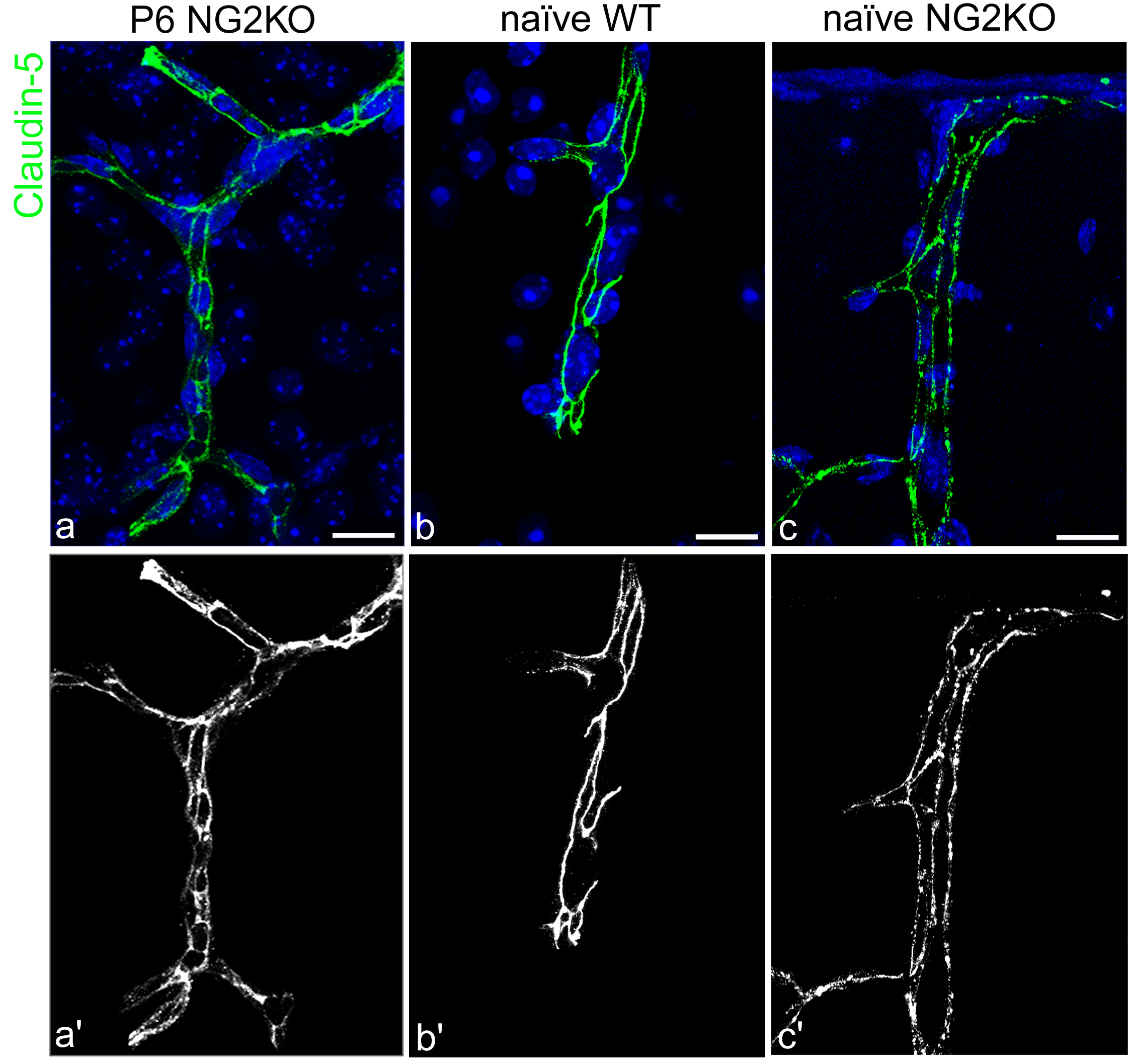

Supplement: S2 Fig — a-c, a’-c’ Comparison of claudin-5 staining patterns between NG2KO mice, both at P6 (a) and in adulthood (c), with adult, naïve WT mice (b). a’, b’, c’ The binary black and white format of the same images shown in (a, b, c) better demonstrates the differences between claudin-5 junctional patterns. Nuclear counterstaining with TO-PRO-3 in (a-c). Scale bars, 10 μm. (TIF) [file pone.0213508.s002.tif]

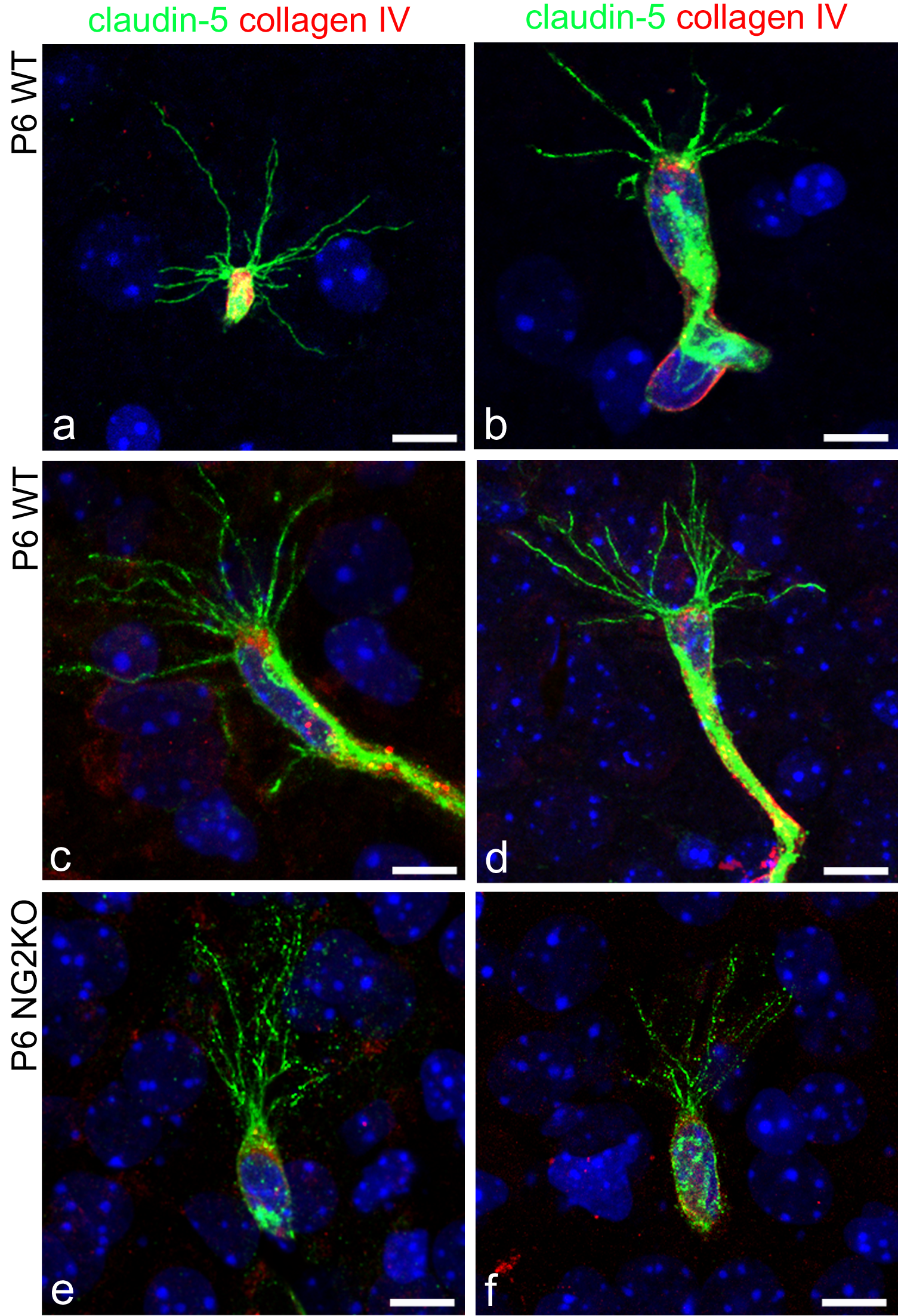

Supplement: S3 Fig — a-f Representative confocal images of vessel sprouts double-immunolabelled for claudin-5 and collagen IV. a-d In P6 WT, endothelial stalk and tip cells show a strong, diffuse claudin-5 staining, which also reveals typical filopodial extensions. e, f In P6 NG2KO mice, sprouting endothelial tip cells show a punctate claudin-5 staining; note the reduced collagen IV staining between WT (a-d) and NG2KO sprouts (e, f). Nuclear counterstaining with TO-PRO-3. Scale bars, a-f 10 μm. (TIF) [file pone.0213508.s003.tif]
